# Hydrostatic sea-level rise inundation impacts on *ahu* and harbors of Rapa Nui (Easter Island)

**DOI:** 10.1038/s41598-026-45195-9

**Published:** 2026-03-22

**Authors:** Noah Paoa, Charles H. Fletcher, Matthew Barbee, Tiffany R. Anderson, Shellie Habel, Gabriel Wilkins Riroroko, Sebastián Pakarati Trengove

**Affiliations:** 1 Department of Earth Sciences, School of Ocean and Earth Science and Technology, University of Hawaiʻi at Mānoa, 1680 East‑West Rd, Honolulu, HI USA; 2 Departamento de Obras, Municipalidad de Rapa Nui, Atamu Tekena, Rapa Nui, Chile; 3 Secretaría Técnica de Patrimonio Rapa Nui, Hotu Matu’a, Rapa Nui, Chile

**Keywords:** Sea-level rise impacts, Hydrostatic modeling, Rapa Nui archaeology, Sea-level rise adaptation, Coastal cultural heritage, *ahu* and *moai* threatened, Climate sciences, Developing world, Environmental sciences, Environmental studies, Geography, Geography, Natural hazards, Ocean sciences

## Abstract

Sea-level rise (SLR) threatens coastal communities, infrastructure, and cultural heritage worldwide, with small islands among the most vulnerable. Rapa Nui, renowned for its *ahu* and approximately 1,000 *moai* statues, faces dual risks: damage to irreplaceable cultural sites and disruption of modern harbors, both fundamental for maintaining cultural continuity and sustaining the island’s economy. This study evaluates the impacts of SLR on Rapa Nui’s coastal heritage and infrastructure using a probability-based hydrostatic inundation model. We establish present-day mean higher high water (MHHW) and the 1% annual exceedance probability (AEP) water level as reference baselines and simulate inundation extents at incremental SLR scenarios up to 3.9 m. Results indicate that four *ahu* are already impacted by present-day sea levels, and eight more are projected to be impacted by 2080. In the same timeframe, harbors are projected to be fully submerged under MHHW conditions. Comparisons between observed and modeled impacts further suggest that hydrostatic modeling inherently underestimates risk in Rapa Nui’s high-energy coastal environment. These results underscore the need for proactive adaptation strategies that integrate cultural heritage preservation with coastal resilience planning and improved hydrodynamic modeling to safeguard Rapa Nui’s identity, economy, and long-term sustainability.

**Supplementary Information:**

The online version contains supplementary material available at 10.1038/s41598-026-45195-9.

## Introduction

Humans have undisputedly warmed the atmosphere, land and ocean, causing, among other issues, a permanent and irreversible rise of sea level^1^. The Intergovernmental Panel on Climate Change (IPCC) has consistently found that the average rate of sea-level rise (SLR) has increased since the beginning of the last century and has continuously accelerated in the last 50 years^2^. The IPCC projects that global sea level could rise 1.01 m by 2100 from a 1995–2014 baseline. However, due to large uncertainties regarding ice sheet dynamics, global SLR approaching 2 m by 2100 and 5 m by 2150 is physically plausible^1^. Moreover, even in the absence of additional deep ocean warming, 4 m of SLR is inevitable due to West Antarctic Ice Sheet collapse^3^. Additionally, by 2100, extreme sea-level events that historically occurred approximately once per century, are projected to occur annually at many locations worldwide^4^. Beyond 2100, sea level is projected to continue rising for centuries and will remain elevated for thousands of years^2^.

Humanity has historically settled near coastlines^5,6^, especially on oceanic islands. Consequently, SLR not only threatens the safety of contemporary coastal populations and properties but also the irreplaceable, tangible heritage of past coastal societies^7–9^. Beyond the immediate physical impacts of SLR on coastal assets, impacts on domestic and international tourism, stimulated and supported by these assets, may also profoundly affect the community’s socioeconomic stability^6,10^. In many small island contexts, the threats of SLR are especially evident as they are often isolated and frequently lack sufficient resources or infrastructure to effectively adapt to the changing environment^11^. Therefore, examining and understanding the impacts of SLR on cultural assets and critical coastal infrastructure in such regions is imperative for developing effective adaptation strategies, assessing economic implications and ensuring long-term resilience.

Rapa Nui, also widely known as Easter Island, is a compelling example of an island facing such challenges. Located in the southeastern Pacific Ocean, Rapa Nui is famed for its nearly 1,000 monumental *moai*, multi-ton anthropomorphic statues, many of which were erected along the island’s coastline, on megalithic stone platforms called *ahu*^12,13^. This heritage manifests its importance to the local community in two principal ways. First, *ahu* and *moai* are fundamental in reaffirming identity and supporting the revitalization of cultural practices^14,15^ that were disrupted or lost due to hardships, such as slavery and the introduction of diseases, stemming from European contacts starting in the early 1700s^16,17^. Second, in 1995, the United Nations Educational, Scientific and Cultural Organization recognized the Rapa Nui National Park as the first cultural World Heritage Site (WHS) in Polynesia due to its outstanding universal value to humanity^18^. This recognition solidified tourism as the island’s primary economic driver, attracting tens of thousands of visitors annually^19^. However, SLR poses a two-fold threat to Rapa Nui: (1) it directly impacts the island’s coastal cultural assets, which are essential to the modern Rapa Nui cultural identity and key drivers of the tourism-based economy; and (2) it jeopardizes modern coastal infrastructure, such as harbors, which are vital for sheltering local subsistence fishing vessels, disembarking cruise ships, and unloading goods that are critical for the local community and the tourism industry alike.

The IPCC projects that, by the end of the century, sea level in Rapa Nui will reach 0.32–0.70 m to 0.48–0.94 m relative to a 1995–2014 baseline under the intermediate and very-high greenhouse gas emission pathways (SSP2-4.5 and SSP5-8.5, respectively)^1^. These projections are on the low end of the spectrum given the future sea-level uncertainties associated with ice sheet processes^20–23^. Despite examinations of extreme sea levels in Rapa Nui^24,25^ and various preservation efforts for cultural assets in the context of climate change^26–30^, SLR-related modeling and risk mapping of coastal cultural assets and crucial coastal infrastructure remain remarkably scarce. This is particularly concerning given that SLR potentially poses greater devastating impacts on island communities^8^. Furthermore, tide gauge data analyses and field surveys suggest that extreme sea levels at Rapa Nui occur far more frequently than previously thought, arising from a combination of storm surges, seiches, and high tides linked to atmospheric rivers^24^.

Here, we assess the extent of SLR-induced inundation under hydrostatic conditions using a combination of empirical observations and probability-based hydrostatic inundation simulation. We use the local tide gauge data to determine present-day mean higher high water (MHHW) as reference for modelling hypothetical increments of sea level up to 3.9 m in 30 cm intervals. Additionally, we project the inundation of extreme water levels defined as the 1% annual exceedance probability (AEP) above MHHW to assess exceptionally high sea-level occurrences. To estimate the timing of the inundation and its impacts, we employ SLR timing projections developed by Sweet et al. (2022), which are derived from downscaling the global SLR scenarios from the IPCC AR6 and incorporating the non-uniform contributions of ice sheet melt^31^. According to these projections, sea level around Rapa Nui could reach 1.2 m as early as 2080 under the High scenario, or between 2110 and 2120 under the Intermediate scenario^31^. Under the High scenario, sea level may reach up to 3 m by 2130^31^. The extent of inundation is superimposed on geospatial layers containing the locations of *ahu* to identify those affected by the inundation. Additionally, we assess the susceptibility of the island’s harbors to inundation by determining the timing and elevation at which they will experience submersion. The final products include geospatial layers of inundation extent and inundated *ahu*. Notably, hydrostatic models do not account for dynamic processes like run-up or storm surge, thus the resulting inundation extents should be interpreted as conservative, minimum estimates of potential exposure. These layers can be made available for use by community and local government entities towards planning, developing adaptation strategies, and identifying high-priority locations where detailed surveys and computationally intensive hydrodynamic modeling would be most valuable.

## Background

**Fig. 1:**
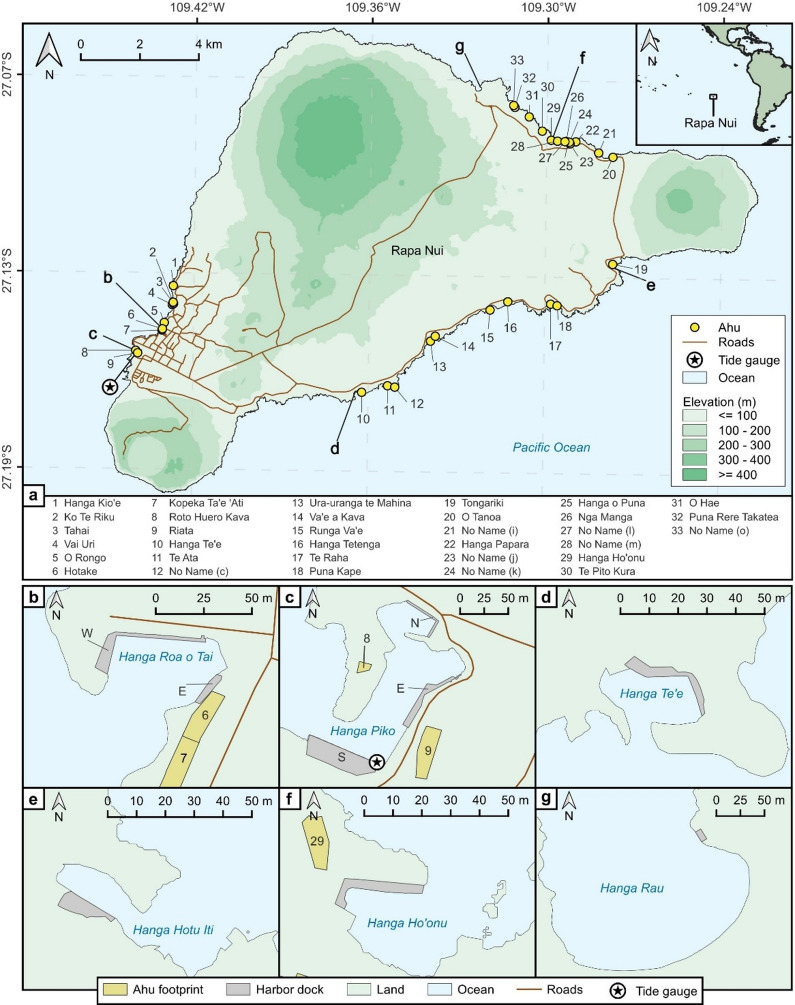
Geographical context of Rapa Nui, showing the distribution of *ahu* and harbors. In panel **a**, the inset map identifies Rapa Nui in reference to Central and South America. The locations of *ahu* are identified with yellow circles and the tide gauge with a black star. Numbers attached to the yellow circles identify the name of each *ahu*. The harbors are labeled with letters corresponding to detailed views in panels **b**–**g**: Hanga Roa o Tai (**b**), Hanga Piko (**c**), Hanga Te’e (**d**), Hanga Hotu Iti (**e**), Hanga Ho’onu (**f**), and Hanga Rau (**g**). In panels **b**–**g**, *ahu* footprints and harbor docks are shown as yellow and grey polygons, respectively. When several docks exist in a harbor, they are distinguished by letters based on the cardinal direction of their location from the center of the harbor. Panel **a** illustrates a subset of the surveyed *ahu*, impacted by one or more of the SLR scenarios; for a list of all surveyed *ahu*, see Supplementary Fig. S1. (Figure created using QGIS 3.40.5; https://qgis.org/)

Rapa Nui, a roughly 164 km^2^ island on the Nazca Plate in the southeast Pacific Ocean, lies approximately 3,700 km off the coast of South America, just south of the Tropic of Capricorn at 27.12° S (Fig. 1a). The island is the product of hotspot volcanism between 0.78 − 0.11 Ma, formed through the coalescence of three volcanoes: Rano Kau, Poike, and Maunga Terevaka^32–34^. Due to plate tectonics, the island is moving east at approximately 0.06 m per year (m yr⁻¹) and subsiding at a rate between 0.001 and 0.004 m yr⁻¹^35,36^. The coastline of Rapa Nui is mainly rocky due to its young age^37,38^. Rapa Nui is triangular in shape, with coastal topography characterized by high cliffs along its corners and edges that descend to lower elevations. Although coral reef structures in incipient stages of development occur along portions of the coastline, the island lacks a continuous fringing reef system^39^.

The island has six harbors: Hanga Roa o Tai and Hanga Piko on the west coast (Fig. 1b, c), Hanga Te’e and Hanga Hotu Iti on the southeast coast (Fig. 1d, e), and Hanga Rau and Hanga Ho’onu on the northeast coast (Fig. 1f-g). Hanga Piko (Fig. 1c) is the island’s largest harbor and the only one capable of supporting cargo unloading operations. While cruise ship passengers may disembark at other harbors, Hanga Piko serves as the primary point for both passenger arrivals and the offloading of imported goods. Its relatively larger size makes this possible, though it remains too small to accommodate direct docking of large vessels; passengers arrive via tender boats, and goods are transferred using small barges when the ocean conditions allow it^40^. This harbor also contains Rapa Nui’s only tide gauge. All harbors moor local subsistence fishing vessels, except for Hanga Rau. The prominent swell direction is from the SSW and SW^41^ as the swells are generated by extratropical cyclones moving between 40–60°S^24^. The island’s triangular shape allows Hanga Rau (Fig. 1g) to serve as a secondary disembarkation point for cruise ships when the prominent swells are active. The ocean environment in Rapa Nui exhibits a mixed tidal regime with a maximum amplitude of approximately 0.8 m during spring tides and 0.3 m during neap tides^24^.

**Fig. 2:**
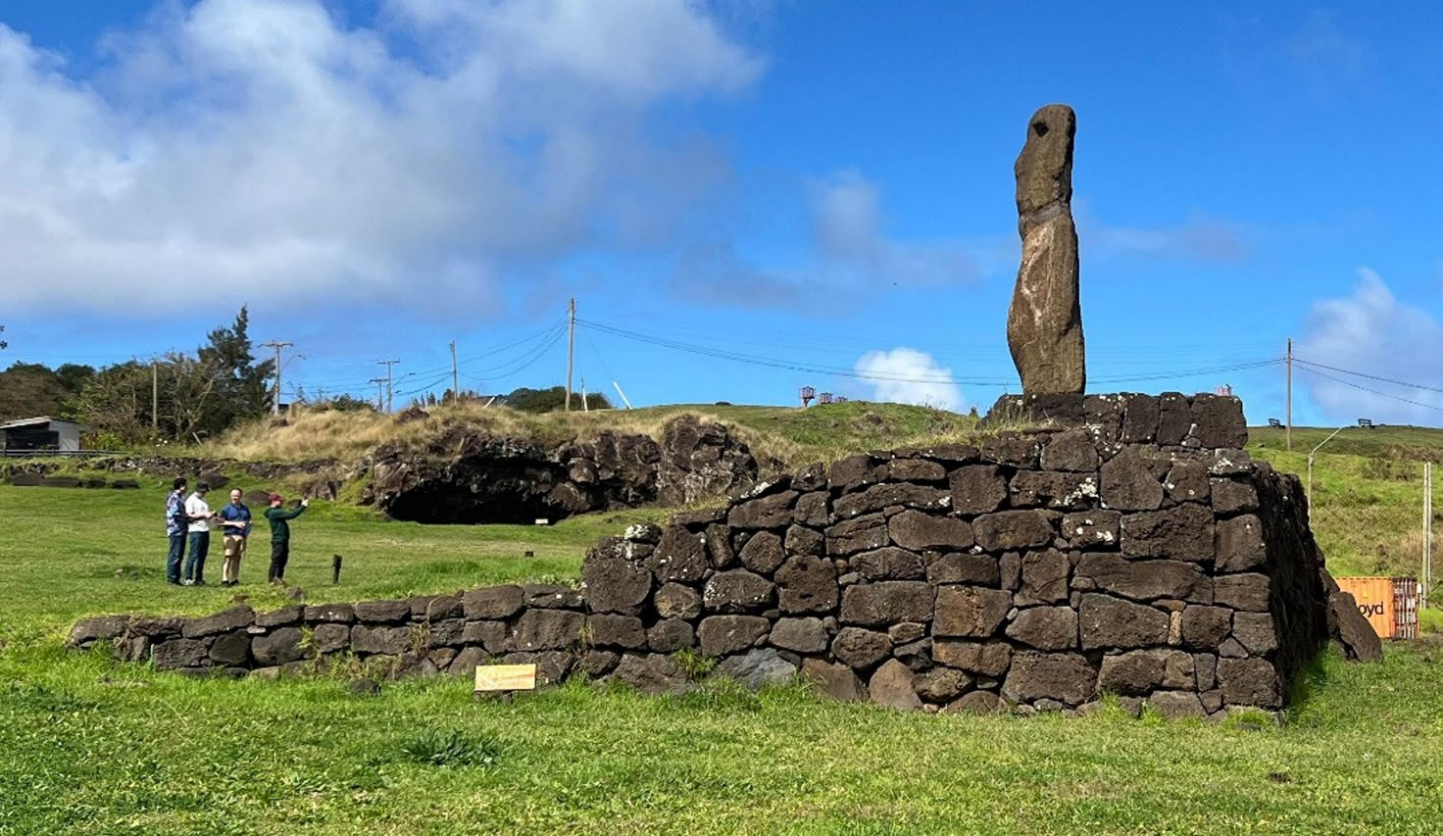
Side view of Ahu Riata (*ahu* no. 9) illustrating the front sloping ramp and the vertical retaining wall.

The coastline of Rapa Nui features over 200 *ahu*: ceremonial stone structures on some of which *moai* were erected to perpetuate the memory of ancestors^16,42,43^. These structures have been extensively studied by researchers^16,43–45^ and 14 of them have been restored. Typically, an *ahu* consists of an elongated and elevated stone platform fronted by an open plaza^45^. Most were constructed parallel to the coast, featuring a seaward-facing retaining wall—which can exceed 5 m in height—and a sloping ramp on the inland side^16,44^(Fig. 2). These structures can significantly alter the coastal landscape, often making it difficult to discern the natural underlying topography from the man-made construction. For this analysis, we included all *ahu*, irrespective of their specific typology, located below the 8 m contour line relative to local mean sea level (LMSL) (for a list of all surveyed *ahu*, see Supplementary Fig. S1).

**Fig. 3:**
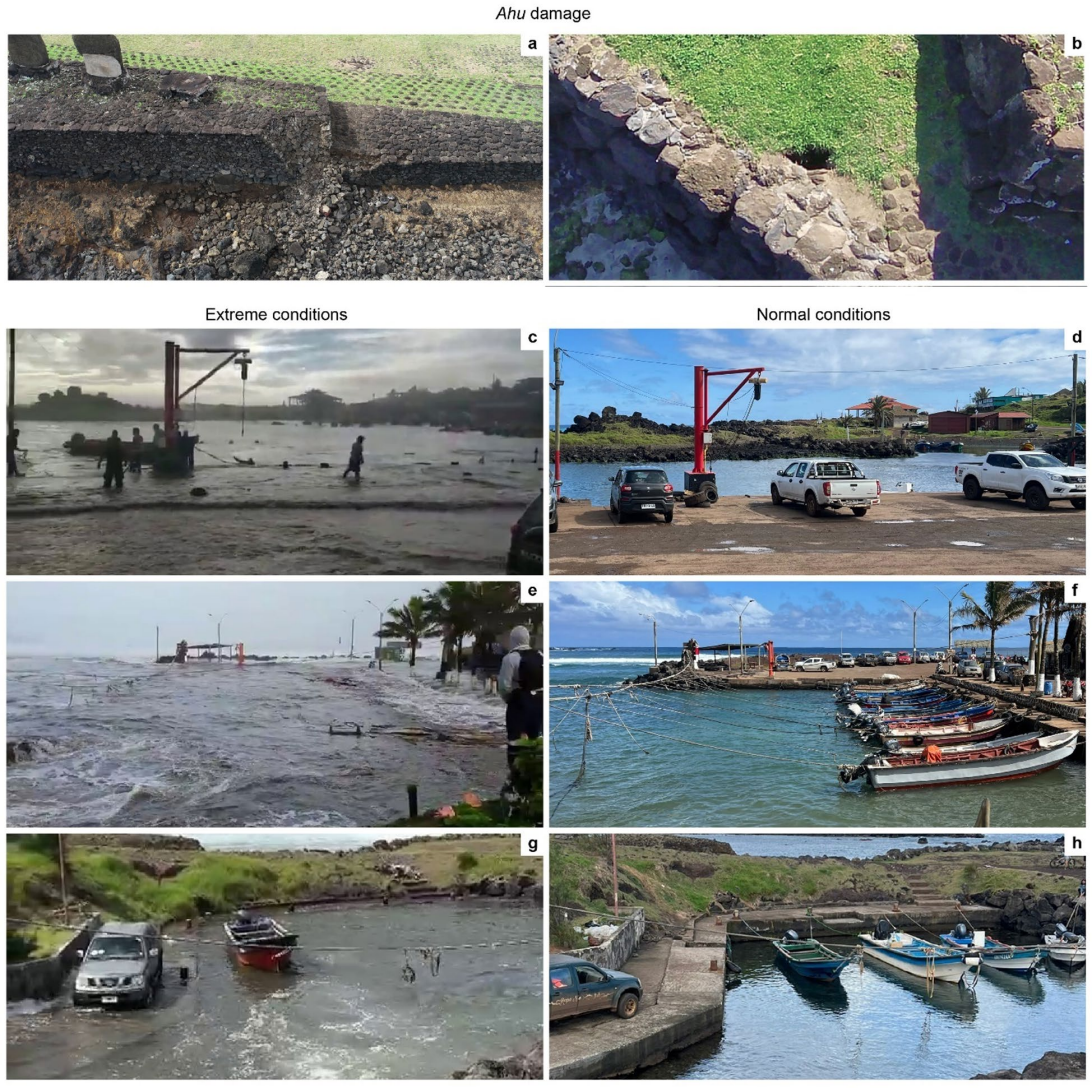
Impacts of extreme oceanographic conditions on Rapa Nui. Panels **a** and **b** show damage to *ahu* caused by extreme swell events: (**a**) partial collapse of the retaining wall of Ahu Vai Uri (*ahu* no. 4) (May 28, 2021) and (**b**) map view of sinkhole adjacent to the retaining wall of Ahu Kopeka Ta’e ‘Ati (*ahu* no. 7) (August 5, 2025). Panels (**c**-**h**) compare extreme and normal sea-level conditions at three harbors: Hanga Piko (**c**, **d**) and Hanga Roa o Tai (**e**, **f**) during storm surge conditions and Hanga Te’e (**g**, **h**) during a swell event. Images in panels **a**, **c**, **e**, and **g** are reproduced with permission from Albert Castel Rapu, Javier Ika Acuña, Cristián Moreno Pakarati, and Hector Maldonado Solís.

Despite their cultural and economic importance, both the *ahu* and harbor infrastructure of Rapa Nui are increasingly vulnerable to extreme oceanographic conditions. Over the past five years, extreme oceanographic conditions have damaged coastal *ahu*, partially collapsed a retaining wall (Fig. 3a) and caused sinkholes (Fig. 3b), thereby threatening their structural integrity. During these events, several of the island’s harbors also experienced inundation, some of which have been documented and analyzed^24^. For instance, during May 2020, extreme sea levels led to the complete inundation of the N and E docks of Hanga Piko (Fig. 3c). The water levels at the N and E docks during this event were decimeters higher than those recorded by the tide gauge located on the S dock, closer to the harbor opening, but within the same harbor site^24^. This discrepancy was attributed to the combined influence of local seiches and the constructive superposition of storm surges and high tides. More recently, complete inundation was also documented at Hanga Roa o Tai in May 2021 (Fig. 3e) and Hanga Te’e in April 2025 (Fig. 3g). In Fig. 3, panels d, f and h show normal sea levels at Hanga Piko, Hanga Roa o Tai and Hanga Te’e, respectively, for comparison.

## Results

**Fig. 4:**
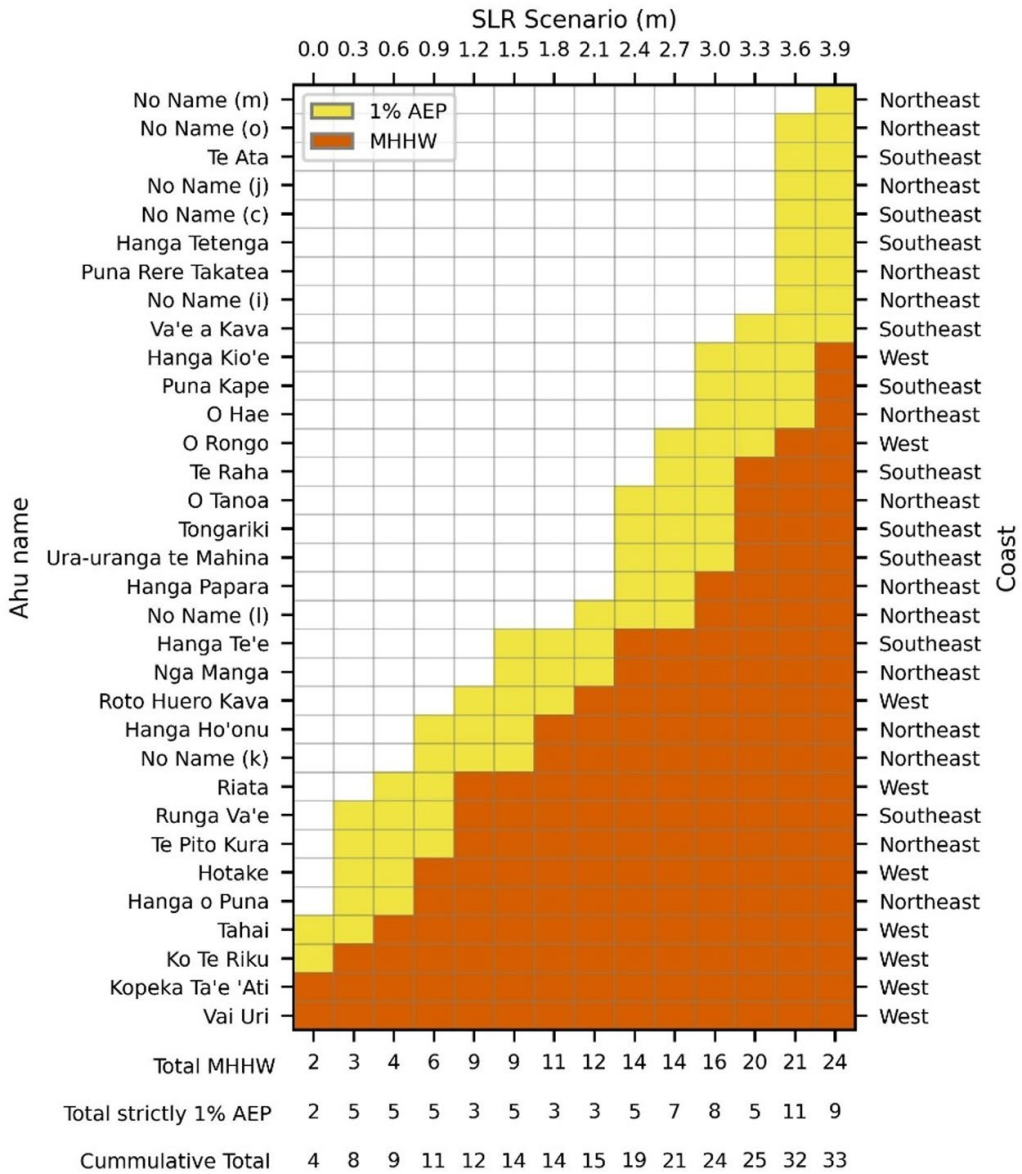
Rapa Nui *ahu* impacted by SLR scenarios and tidal inundation thresholds. The heatmap shows individual *ahu* (left y-axis) ordered by the earliest inundation occurrence due to SLR (top x-axis). Shading indicates whether inundation occurs at MHHW (orange) or at the 1% AEP level (yellow). The coastal location of each *ahu* is listed on the right y-axis. The bottom x-axis provides a cumulative count of affected *ahu* per SLR increment and specifies the number of *ahu* affected at MHHW versus at strictly the 1% AEP level.

**Fig. 5:**
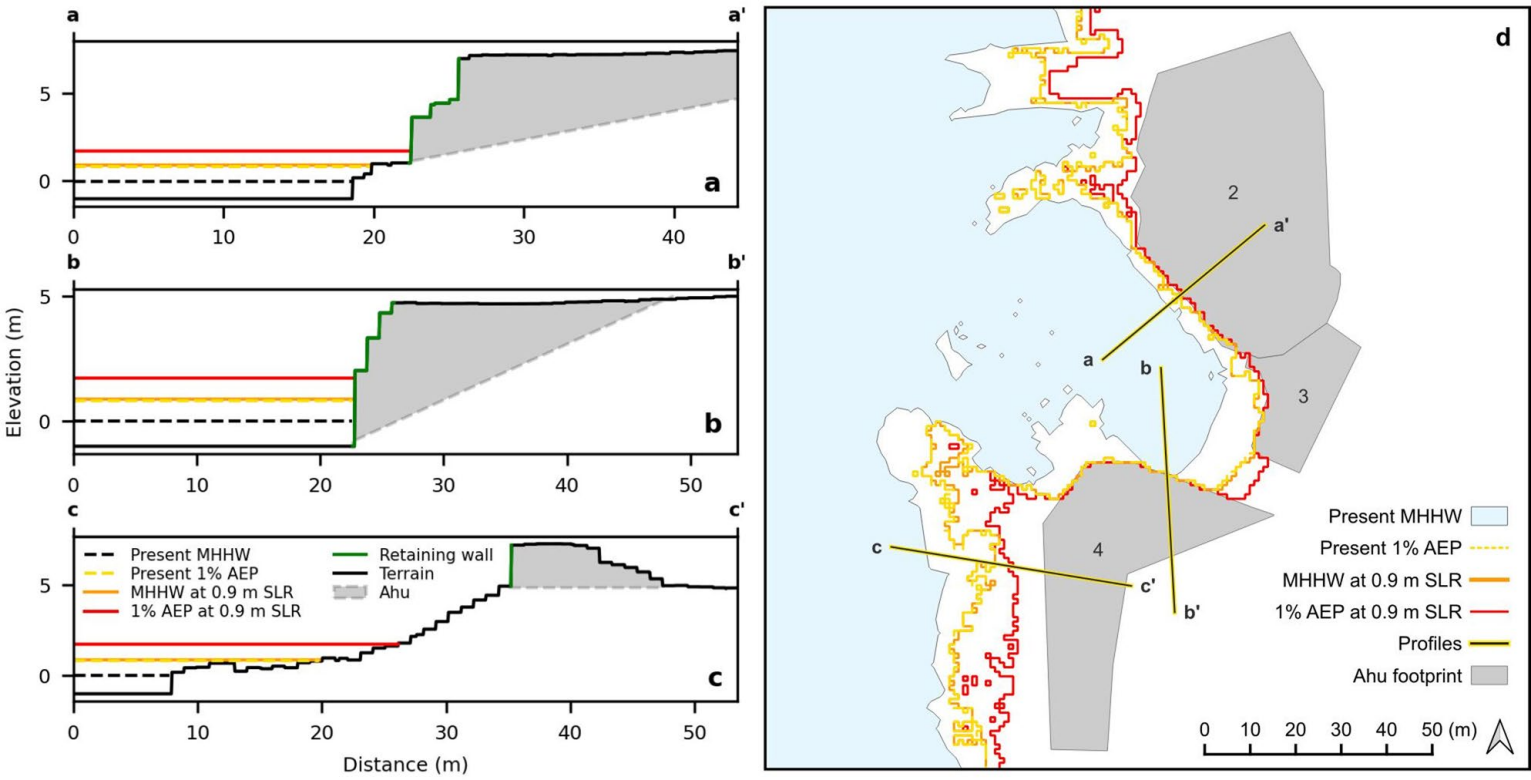
Site map and cross-sections of Ahu Ko Te Riku (*ahu* no. 2) (**a**) and Ahu Vai Uri (*ahu* no. 4) (**b**, **c**). *Ahu* no. 3 corresponds to Ahu Tahai, not shown in cross-section. Panel **d** is the site map showing the cross-section locations in relation to the *ahu* footprints (grey polygons). The numbers within the polygons correspond to the *ahu* list in Fig. 1a. In all panels, projected water levels are shown for a 1% AEP level at 0.9 m of SLR (red line), MHHW at 0.9 m of SLR (orange line) and present-day 1% AEP level (dashed yellow line). The present-day MHHW is represented by a dashed black line (panels **a**-**c**), and the blue area (panel **d**). In the cross-sections, the black line denotes terrain elevation, the green line indicates the *ahu* retaining walls and the grey shaded area represents the *ahu* structure, with a dashed base denoting unknown underlying terrain. See Supplementary Figs. S4–S6 for comparisons of outcomes across probability values used in confidence mapping. (Panel **d** was created using QGIS 3.40.5; https://qgis.org/)

Hydrostatic modeling is a common methodology for assessing the impacts of SLR on coastal cultural assets^6,9,46–48^. In this study, we applied a probability-based hydrostatic approach to assess the risk of coastal flooding to Rapa Nui’s *ahu* and harbors. The local MHHW and 1% AEP level, stand at 0.295 m and 1.127 m above LMSL, respectively. These benchmarks serve as reference points throughout the results section, enabling readers to contextualize our findings and to compare them with in-situ observations and results reported in other scientific publications. We simulated inundation for SLR scenarios ranging from present-day LMSL up to 3.9 m in 30 cm increments, mapping inundation extents at an 80% confidence level to account for elevation data uncertainty. This value indicates that we are 80% confident that the area within the projected inundation extent will be under water, or conversely, we are 20% confident that the area beyond the inundation extent is expected to be dry. Although 30 cm increments are finer than the minimum statistically significant SLR increment at 95% confidence (see *Uncertainties and Assumptions* section), they are used here to (1) maintain consistency with commonly reported SLR scenarios across both metric and imperial reporting conventions, and (2) allow aggregation into statistically robust increments (≥ 0.6 m) where required for decision-making. Due to the large number of *ahu* and large study area, illustrations of SLR inundation extent and their intersection with *ahu* footprints are not presented for all locations. Instead, we show synthesized results in a heat map presented in Fig. 4 and an example of mapped inundation extent and cross-section water level impacts at one coastal site in Fig. 5.

### *Ahu*

Our initial survey identified 67 *ahu* located partially or fully within the 8 m elevation contour relative to LMSL, including 8 restored *ahu* (a list of *ahu* names and locations is available in Supplementary Fig S1). These *ahu* are distributed across the west (11), northeast (25), and southeast (31) coasts. Of these *ahu*, 33 (those shown in Fig. 1a) are projected to intersect the inundation extent in at least one SLR scenario. Figure 4 lists the impacted *ahu* ranked according to their relative inundation threshold; the lowest SLR scenario at which each *ahu* footprint becomes intersected by the inundation extent. Naturally, once an *ahu* is intersected by the inundation extent at a given sea level, it remains highlighted in all subsequent SLR scenarios as the intersection between the inundation extent and the *ahu* footprint will only enlarge. Since all *ahu* reached by MHHW are also reached by the 1% AEP level, Fig. 4 explicitly identifies *ahu* that are impacted strictly by the 1% AEP (and not MHHW).

Our model projects two *ahu* intersected by the present-day MHHW elevation contour (Ahu Vai Uri and Ahu Kopeka Ta’e ‘Ati), and two additional *ahu* intersected by the present-day 1% AEP level inundation extent (Ahu Ko Te Riku and Ahu Tahai). The number of *ahu* reached by the inundation extent under MHHW conditions generally increases by one to three for each 0.3 m SLR increment. The exceptions are the 1.5 m and 2.7 m SLR scenarios (no increase), and the 3.3 m SLR scenarios which shows an increase of four *ahu*. In every SLR scenario the 1% AEP level reaches an additional two to eleven *ahu* beyond those intersected by MHHW, typically impacting them two to three SLR increments earlier. At the highest scenario (3.9 m SLR), a total of 33 *ahu* are identified: 24 under MHHW conditions and an additional 9 under 1% AEP conditions. The *ahu* projected to be affected earliest are predominantly located on the west coast, followed by the northeast and southeast coasts. Within the first 0.9 m of SLR, all *ahu* affected at MHHW are on the west coast. By 2.1 m of SLR, all affected *ahu* are on the west and northeast coasts, with the sole exception of Ahu Runga Va’e on the south coast. Ahu Tongariki, the largest *ahu* in Rapa Nui, is intersected by the 1% AEP level inundation extent at 2.4 m of SLR and by the MHHW inundation at 3.3 m of SLR. Nearly all projected *ahu* intersections occur through topographically connected flooding. The only two exceptions are Ahu Tongariki (*ahu* no. 19) and Ahu Te Pito Kura (*ahu* no. 30), which, in addition to topographically connected flooding on their backwall, are projected to experience topographically disconnected flooding associated with groundwater emergence on their inland perimeter, highlighting a secondary but important exposure pathway that may require different adaptation strategies. Groundwater-driven inundation at Ahu Tongariki (*ahu* no. 19) is first projected at 3.3 m of SLR and persists under higher SLR scenarios (3.6 m and 3.9 m). Similarly, Ahu Te Pito Kura (*ahu* no. 30) will experience topographically disconnected flooding in addition to topographically connected flooding, but only at SLR scenario 2.7 m.

The geomorphological setting of individual *ahu* dictates its unique vulnerability to coastal inundation. Figure 5 provides a detailed analysis of the extent of inundation for MHHW and the 1% AEP level at both present-day sea level and with an additional 0.9 m of SLR for a coastline section containing three adjacent *ahu*: Ahu Ko Te Riku (*ahu* no. 2), Ahu Tahai (*ahu* no. 3) and Ahu Vai Uri (*ahu* no. 4). Cross-sectional profiles (Fig. 5a-c) and a map view (Fig. 5d) illustrate the vertical position of the four sea-level scenarios relative to the terrain and the horizontal inundation extent, respectively. The results highlight local variations in flood patterns. In some areas, higher sea levels lead to greater inland water penetration, whereas in other areas, particularly near the *ahu*, the steeper terrain constrains the horizontal extent of inundation. Notably, the projected inundation extent for the present-day 1% AEP event is nearly identical to that of a MHHW tide with 0.9 m of SLR. The inundation extent lines of some scenarios overlap in several segments, particularly along steep sections adjacent to the *ahu*, whereas in other areas they diverge, reflecting lower gradient slopes and larger horizontal differences between SLR scenarios (Fig. 5d). Modeling suggests that the northern wall of Ahu Vai Uri (*ahu* no. 4) is already impacted at present-day MHHW (Figs. 4 and 5b and d), while Ahu Ko Te Riku (*ahu* no. 2) and Ahu Tahai (*ahu* no. 3) are impacted at the present-day 1% AEP level. All *ahu* at this site are intersected on their bay-facing wall rather than along coastline sections outside the bay (Fig. 5d). As with all elevation-based inundation mapping, uncertainty translates into greater horizontal variability in low-gradient terrain than in steep terrain. Consequently, it must be considered, especially in low-gradient terrain, that the inundation extents presented are probabilistic inundation zones (80% confidence) rather than sharp boundaries.

### Harbors

**Fig. 6:**
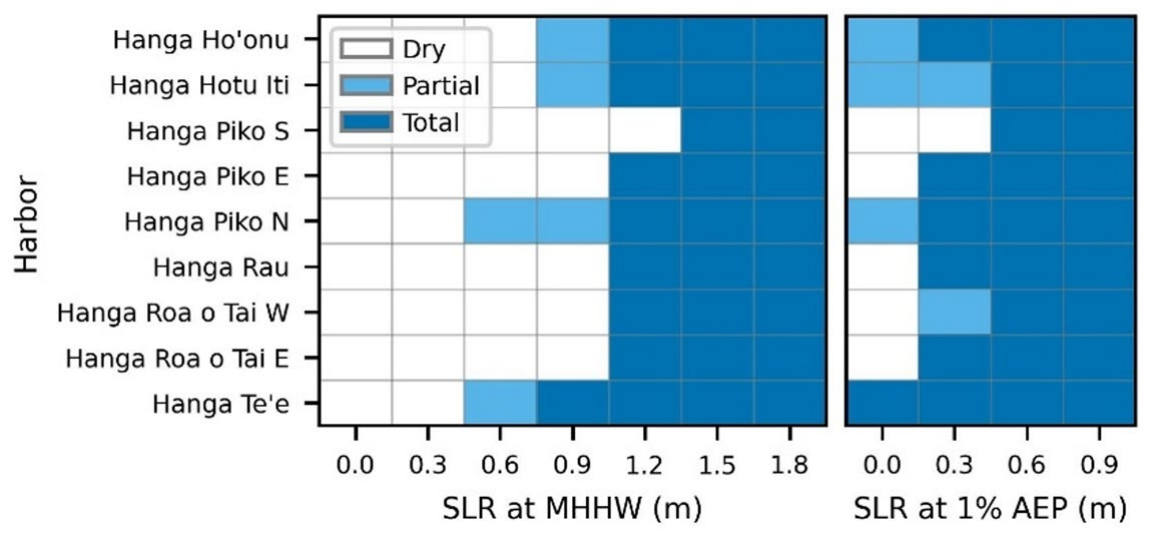
Harbor dock submersion thresholds under MHHW and 1% AEP conditions. The figure compares inundation thresholds at MHHW (left panel) and 1% AEP levels (right panel). Each harbor is listed on the shared y-axis. Colors indicate the level of impact at a given SLR scenario: white for dry, light blue for partial inundation and dark blue for total inundation. For a map view of inundation extents, see Supplementary Fig. S3.

Like the *ahu*, Rapa Nui’s harbors are engineered structures whose vulnerability to SLR is determined by their geomorphological setting, their structural design and their alteration of the local topography. Harbor docks were classified as partially or totally submerged based on the proportion of their footprint covered by the extent of flooding. Our inundation modeling shows that the harbors are highly vulnerable to SLR, with important differences between MHHW conditions and 1% AEP events (Fig. 6). Under MHHW conditions, several harbor docks show partial flooding at 0.6–0.9 m of SLR and all except the south dock of Hanga Piko, are projected to be fully submerged at 1.2 m of SLR. Inundation during a 1% AEP event is more severe. At present-day sea level, several harbors experience partial or total flooding, and at 0.3 m of SLR all harbors are projected to experience total submersion in at least one of their docks (where applicable). The exception is Hanga Hotu Iti, which is projected to experience only partial flooding. Notably, the inundation projected for a 1% AEP event at 0.6 m of SLR is nearly equivalent to that of the MHHW level at 1.2 m of SLR, except at the south dock of Hanga Piko. Two docks are notable exceptions to the general trends: Hanga Te’e and the south dock of Hanga Piko. Hanga Te’e is the first harbor to be fully submerged under MHHW and it is shown to experience total submersion under 1% AEP condition at present-day sea levels. The south dock of Hanga Piko is the last one to be fully submerged in both cases and is not projected to experience even partial submersion until sea level rises 1.5 m over present-day MHHW or with 0.6 m SLR under 1% AEP conditions. SLR scenarios beyond 1.8 m under MHHW conditions and 0.9 m under 1% AEP events are omitted as they provide no additional insight. No harbor docks were identified as being threatened by groundwater inundation. A map view of the inundation extents for each harbor at key SLR scenarios is provided in Supplementary Fig. S3.

## Discussion

Our analysis of *ahu*, summarized in Fig. 4, reveals a hierarchy of vulnerability among Rapa Nui’s coastal *ahu.* Ranking *ahu* by their inundation threshold provides an objective basis for prioritizing sites for SLR-impact mitigation or further site-specific investigations. It is not surprising that some *ahu* are found within the inundation extent even at present-day MHHW levels. This is consistent with observed impacts to Ahu Vai Uri (Fig. 3a) and Ahu Kopeka Ta’e ‘Ati (Fig. 3b) during past extreme oceanographic conditions, and with our model results, which show that the *ahu* intersected by present-day inundation coincide with the *ahu* that have already experienced damage due to direct wave action. In such cases, the objective is not to highlight their vulnerability to higher sea levels, but rather to emphasize that these impacts are already a present-day reality, to corroborate their priority for preservation, and to exemplify the risks that higher-ranked *ahu* in Fig. 4 will face as sea level rises. Following Sweet et al.‘s (2022) High scenario, 12 *ahu* would be intersected by the extent of inundation by the year 2080 (+ 1.2 m of SLR) and Ahu Tongariki, the largest *ahu* in Rapa Nui, could be reached as soon as the year 2110 (+ 2.4 m of SLR). This projection contrasts with a recent SLR study that estimates Ahu Tongariki’s intersection as early as 2080 when waves are considered^49^, underscoring the critical role of wave action in accurately predicting coastal inundation and the inherent underestimation of potential impacts by hydrostatic models. This limitation is particularly important along Rapa Nui’s west and south coasts, as the prominent swell direction is from the southwest^41^.

The cross-section profiles provide a clearer view of how inundation progressively expands around *ahu* under incremental SLR scenarios. The close correspondence between the present-day 1% AEP extent and the MHHW extent at + 0.9 m of SLR underscores how contemporary extreme sea levels serve as an analogue for the normal sea levels of the future. In this cross-section, Ahu Vai Uri (*ahu* no. 4) is intersected on its north-facing wall (Fig. 5b). However, the damage documented on this *ahu* following the extreme oceanographic conditions of May of 2021 occurred on the west-facing wall^50^(Fig. 3a). We attribute this discrepancy to the uninterrupted sloping terrain leading up to the west-facing wall (Fig. 5c), rather than to wall orientation relative to the coastline and the prominent swell direction. This interpretation is supported by the fact that Ahu Ko Te Riku (*ahu* no. 2) and Ahu Tahai (*ahu* no. 3), which share the same orientation but are fronted by rocks or have steeper slopes leading up to their retaining wall (Fig. 5d), did not experience noticeable damage. The absence of impacts on the north-facing wall of Ahu Vai Uri suggests that the structure could withstand at least small amounts of SLR, provided that wave energy is no greater than that experienced on its west-facing wall in May of 2021. Although individual *ahu* have unique features and are built on distinctive landscapes, their rock composition and general construction are largely consistent, allowing this inference to be extended to other *ahu*. Moreover, because waves reached Ahu Vai Uri at a location not predicted by the hydrostatic inundation model, it is safe to assume that other *ahu* could be directly impacted by waves earlier than the hydrostatic model projections suggest.

Although not organized by impact threshold, the limited number of harbors allows the heatmap in Fig. 6 to easily identify harbors and individual docks that should be prioritized for adaptation planning under future sea levels. The total inundation of Hanga Te’e and the partial inundation of multiple docks under present-day 1% AEP level, together with total inundation of most docks with only + 0.3 m of SLR, highlights the present-day vulnerability of Rapa Nui harbors, already evident during swell and storm surge events (Fig. 3c-g). These results further indicate that hydrostatic flood models underestimate potential impacts by excluding dynamic processes such as swell, storm surge, and seiches, which can combine to amplify sea levels. An expanded inundation area could be explored by decreasing the confidence level used for hydrostatic modeling, but this approach is not designed to simulate the compounding effects of these processes. Evidence from the May 2020 extreme oceanographic event illustrates this limitation. Photographs and schematics of Hanga Piko^24^ suggest that Ahu Riata, near the east dock (Fig. 1c), was reached by ocean waters under present-day extreme oceanographic conditions, whereas our simulations indicate inundation at that location only under 1% AEP levels with + 0.6 m SLR or under MHHW with + 1.2 m of SLR. Despite this discrepancy in timing, the modeled flood extents are comparable to those observed when sea level was estimated at 1.4–1.7 m above LMSL^24^. This range is approximately equivalent to our MHHW level at + 1.2–1.5 m SLR or 1% AEP level at + 0.6–0.9 m SLR (see Supplementary Fig. S3). The elevated sea levels during this event were attributed to constructive superpositions of seiches, storm surges and high tides^24^, underscoring the importance of hydrodynamic approaches for assessing SLR impacts. Waves further exacerbate the risks to harbors by enhancing flooding and potentially overtopping protective barriers, thereby reducing the security of mooring areas and facilities for unloading goods and cruise ship passengers. Assuming the sea levels observed at Hanga Piko (Fig. 3c, d) and Hanga Roa o Tai (Fig. 3e, f) represent exceptional events, and following Sweet et al.’s (2022) projections, we estimate that all docks (except for the south dock of Hanga Piko) could be completely inundated under MHHW by 2080, and all harbors will experience total inundation under 1% AEP levels as early as 2050 in at least one (where appropriate) of their docks. Importantly, extreme events already observed provide real-world analogues of future tidal conditions, offering insight into how critical infrastructure may respond to projected sea levels.

The *ahu* and harbors of Rapa Nui are irreplaceable elements of the island’s cultural identity and economic sustainability. *Ahu*, and their associated *moai*, embody multiple layers of cultural value, reinforcing their role in community life, defining the island’s coastal landscape and serving as sites of interest for tourism. Beyond their role in the economy, these monuments sustain intangible heritage by reaffirming community identity, nurturing spiritual connection with ancestors, and supporting revitalization of cultural practices^14,15^. Although more recent in origin, harbors are likewise essential to Rapa Nui’s resilience. They provide infrastructure for importing goods, receiving cruise ships and protecting subsistence fishing vessels that sustain both local livelihoods and the tourism economy. Moreover, they support fishing practices that remain integral to Rapa Nui culture. Preserving *ahu* and harbors could, therefore, prove vital not only for safeguarding cultural identity but also for ensuring economic wellbeing. Physical damage to *ahu* and harbors could threaten not only their material integrity but, in some contexts, the transmission of cultural meaning, raising urgent concerns regarding preservation and intergenerational equity in both cultural and economic contexts.

While the cultural value of Rapa Nui’s coastal heritage threatened by SLR is incalculable, the island’s social wellbeing and economic viability are structurally dependent on tourism, which directly or indirectly employs over 90% of the local population^40^. Although widely supported by residents^40^, this industry intensifies pressure on environmental resources, archaeological heritage and the island’s fragile supply chain. The economic model is sustained by a critical logistical dependence on the continent, with the high cost of air freight making maritime transport, and thus the harbors, the essential lifeline for importing construction materials and almost 90% of food products^40,51^. Energy supply is similarly vulnerable, as imported natural gas, used for heating, accounts for about 20% of the island’s energy generation^51^. This near-total reliance on maritime transport underscores the strategic importance of the island’s harbor infrastructure, some of which have already suffered important damage from swell events^40^. Initial assessments suggest that harbor operability will be compromised by SLR and storm-surge-induced inundations by 2100^40^, yet our findings indicate that most harbors are likely to be inundated within decades, and documented observations^24^ suggest that even these projections may be conservative.

The necessity of an integrated management system that links cultural heritage and the tourism sector, guided by clear long-term policies and objectives, has already been recognized^40^. Incorporating SLR into such frameworks is imperative, particularly given that total protection from coastal hazards is not always achievable. In this context, three principal adaptation strategies are generally recognized: accommodation, protection, and managed retreat^52^. Yet in practice, many adaptation measures span multiple categories, and scholars often disagree on their classification^52^. Moreover, their application to cultural and archaeological site management has received limited attention. Accommodation strategies involve accepting coastal change and modifying existing structures to cope with new conditions, while also implementing intensive monitoring and developing appropriate policy frameworks^52^. Such strategies may also encompass community decisions to allow partial or total submergence of heritage when cultural continuity and economic wellbeing can still be maintained. Protection typically relies on hard engineering interventions such as the construction dikes, seawalls, or breakwaters to stabilize the shoreline^52^. Managed retreat accepts that certain structures must eventually be relocated entirely^52^. Ecosystem-based adaptation strategies offer additional alternatives, for example through the establishment of mangroves or coral reefs^52^. However, Rapa Nui’s coral reefs are underdeveloped and do not grow in shallow waters^39^, and the introduction of mangroves could alter the island’s ecology^53,54^. Each of these strategies carries advantages and limitations, underscoring the need for careful evaluation before implementation.

Harbors inherently combine elements of accommodation and protection strategies, and as modern constructions, they can reasonably continue to be modified to address future challenges. Designing harbor infrastructure that remains fully functional under present-day conditions while also withstanding multimeter SLR represents a great challenge. More realistically, interventions will need to be incremental, involving repeated modifications, such as progressively increasing harbor height and structural robustness to match rising sea levels. In addition, careful planning must account for stronger wave loadings, for which wave-dampening structures, such as tetrapods or other energy-dissipating structures, may provide viable protective measures.

*Ahu*, by contrast, present a distinct set of challenges. Although they are man-made, they are also sacred cultural heritage structures representing a past reality and unique construction techniques, demanding special considerations. Adaptation measures may balance cultural integrity with structural resilience, drawing on traditional practices where possible to reinforce their cultural significance and ensure that future generations continue to benefit from them both culturally and economically. However, inundation does not inherently preclude continued cultural connection or touristic value, and the local community may chose allow partial or total submersion of their heritage. Such pathways require deliberate evaluation within locally grounded management frameworks. In this context, it is crucial to recognize that decisions regarding the future of the *ahu* rest with the Rapanui community, regardless of the WHS designation. Historical evidence demonstrates that *ahu* often underwent multiple reconstructions or modifications in response to evolving social dynamics^43,45,55–60^ and potentially the associated environmental changes and ecological conditions. However, outsider narratives frequently frame discontinuities in this process as evidence of “broken” traditions^61^, sometimes attributing blame on the Rapanui themselves and implying that cultural continuity cannot be restored. In reality, these disruptions stemmed in part from colonial encounters, missionary interventions, and settler impositions that undermined genealogical and ritual transmission as *ahu* continued to be modified after the first European encounters^59^. To deny the Rapanui authority over restoration and proper adaptation management today is to perpetuate this colonial dispossession allowing for *ahu* and *moai* to become commodified relics, appreciated primarily as archaeological curiosities and stripped of their deeper cultural and spiritual meaning. Full sovereignty in determining how continuity, repair, and renewal are pursued must remain with the Rapanui community, who may choose to modify *ahu*, their surroundings or relocate cultural assets in ways that mirror past adaptive practices as an attempt to restore the disrupted transmission of traditions.

Our analysis suggests that *ahu* presently threatened during extreme oceanographic conditions may benefit from a combination of accommodation and protection responses. Protection strategies could involve the construction of discreet offshore or coastal barriers between the *ahu* retaining wall and the ocean to dissipate wave energy, thereby maintaining *ahu* structural integrity in the short term. Such barriers would help prevent dislodgment of supporting rocks, erosion of basal sediments, and subsequent collapse of retaining walls or internal sections. A key consideration is whether these structures should emulate traditional construction methods, prioritizing cultural sensitivity and minimizing visual impact, or employing well-established wave-dissipating structures such as tetrapods. Notably, preventive measures of this kind have been implemented in the past^62^. Between 2002 and 2004 rock walls were built to reduce soil erosion supporting Ahu Ura Uranga Te Mahina (*ahu* no. 13) and Ahu Runga Va’e (*ahu* no. 15). Accommodation responses could involve fortifying the lower sections of *ahu* to extend their longevity, for example by replacing basal rocks with larger stones or stabilizing the foundations with modern materials like concrete. Regardless of the chosen approach, interventions must effectively dissipate wave energy and partially or fully stabilize the shore or the *ahu* itself. In light of the results shown in Fig. 4, and recognizing that decisions regarding intervention ultimately rest with the Rapa Nui community, we recommend prioritizing for potential mitigation measures all *ahu* intersected by inundation at or below MHHW under 1.2 m of SLR: Ahu Vai Uri, Ahu Kopeka Ta’e ‘Ati, Ahu Ko Te Riku, Ahu Tahai, Ahu Hanga o Puna, Ahu Hotake, Ahu Te Pito Kura, Ahu Runga Va’e, Ahu Riata. Likewise, we recommend monitoring all *ahu* intersected by the 1% AEP inundation level under 1.2 m SLR: unnamed Ahu (k), Ahu Hanga Ho’onu, and Ahu Roto Huero Kava. However, any modifications risk altering the Rapa Nui’s cultural landscape, potentially compromising its WHS status, and may also prove financially prohibitive^63^.

Crucially, all implemented measures for either modern infrastructure or cultural heritage must be embedded within an ongoing process of monitoring and evaluation, enabling flexibility as environmental conditions and socio-economic needs evolve. Beyond the threats of direct wave impact and eventual inundation, *ahu* and *moai* are already vulnerable to salt spray, erosion of supporting sediments and other impacts associated with SLR^64^. Moreover, *ahu* are not isolated monuments but components of larger complexes, often including crematoria, paved ramps to the sea, coastal wells, and associated inland features^43,64^. If Rapa Nui is to enhance its resilience and sustainably manage its infrastructure and unique heritage in the face of rising seas it is essential to contemplate a flexible and iterative approach, blending accommodation, protection, and, where appropriate, strategic retreat, always supported by continuous monitoring and evaluation. Adaptations will be an ongoing process rather than a one-time intervention, and no single strategy can address the full range of risks. Instead, resilience depends on a carefully balanced combination of approaches, guided by cultural sensitivity, ecological sustainability, and the authority of the Rapanui community in decision-making. The incremental inundation scenarios we have generated allow for policies and management decisions to be updated as new SLR timing projections emerge.

Proactive adaptation offers a unique opportunity to integrate preservation and adaptation with restoration efforts, reaffirming Rapa Nui identity, revitalizing cultural practices and reinforcing ancestral connections. This dynamic vision of heritage management contrasts with static preservation approaches that seek to “freeze” archaeological remains in their current form due to the “broken” tradition of modifying for the *ahu*^61^. Instead, recognize Rapa Nui culture as living, evolving and adaptive, allowing for heritage management to foster a positive feedback cycle in which preserved heritage sustains tourism, tourism generates resources for ongoing heritage management, and heritage management empowers the Rapa Nui community to continue transmitting both tangible and intangible heritage. In this way, intergenerational equity involves not only preventing the loss of cultural assets but also enhancing them, ensuring that future generations inherit living symbols of identity rather than fragmented remnants of the past.

## Methods

### Water level

LMSL and MHHW were calculated from the Rapa Nui tide gauge data, provided by the *Servicio Hydrográfico y Oceanográfico de la Armada de Chile* (Hydrographic and Oceanographic Service of the Chilean Navy), for the period 1995–2014 to align with the baseline of the IPCC’s latest report^1^. Furthermore, we determined the 1% AEP value using the entire tide gauge time series of hourly measurements, which extends from April 1970 until the present. The 1% AEP value indicates the water level that on average has a 1% chance of being equaled or exceeded in any given year^65^. In this context, a 1% AEP level can also be considered as the water level of an event that on average is expected to occur once in 100 years. Following the methodology applied by the United States National Oceanic and Atmospheric Administration (NOAA)^65^, we extracted the annual maximum monthly water level from years with at least four months of data. The monthly timeseries was detrended and we removed seasonal variability using the average seasonal cycles calculated by NOAA specifically for the Rapa Nui tide gauge (available from https://tidesandcurrents.noaa.gov/sltrends/calc_avg_seasonal.htm). Subsequently, we computed the annual water level maxima relative to MHHW and fit them to a generalized extreme value distribution using the genextreme function from the stats module in the SciPy python library. The LMSL value relative to the WGS 84 ellipsoid at the tide gauge location is -3.215 m. The MHHW value is 0.295 ± 0.12 m above LMSL, and the 1% AEP value is 0.832 m above MHHW. For modeling we defined the ocean surface as a single-value raster grid at MHHW and 1% AEP level (+ SLR increment), and used a vertical uncertainty of 0.12 m.

### Digital elevation model (DEM)

A DEM of Rapa Nui’s coast was developed by combining an existing LiDAR dataset with newly collected harbor surveys and a single-value grid. The LiDAR survey was carried out between April and May of 2016, and it was financed by the Secretaría Técnica de Patrimonio Rapa Nui (Technical Secretariat for Rapa Nui Heritage)^66^. LiDAR was collected using a Cessna 172E Monomotor equipped with a Leica ALS60 sensor^66^. The survey’s metadata indicates an average flight altitude of 1500 m, and an average point spacing of 0.25 m. The LiDAR data was vertically referenced to the EGM 2008 geoid. Concurrently, aerial imagery was captured using a RCD105 sensor. Unfortunately, the LiDAR data exhibits elevation discrepancies between flight paths in the near-shore environment, corresponding to differing tidal heights during the various data collection times, and further resulting in ground misclassification of points corresponding to whitewash and sea spray. To address these issues, we used cross-shore profiles to identify and manually remove misclassified LiDAR returns (for details see Supplementary Material, Supplementary Fig. 2). Additionally, we removed vegetation, buildings, and other anthropogenic structures to generate a bare-earth DEM. *Ahu* were preserved as they often seamlessly blend into the landscape, making it difficult to discern between the man-made structure and the underlying topography. Similarly, many rock formations were retained as it is challenging to differentiate between rocks on the soil surface and lava flow outcrops from the aerial imagery. From the classified LiDAR data, we derive a raster grid with a 1 m resolution using the “triangulated irregular network” method in Global Mapper 24.1. We subtracted the EGM 2008 geoid values to obtain WGS84 ellipsoidal elevations, matching the vertical reference of the harbor dock surveys.

To capture structural additions and modifications to harbor docks that occurred after the LiDAR acquisition, we surveyed all harbors docks in January 2025, using Emlid RS2+ Globan Navigation Satellite System receivers and four geodetic monuments: one for the west coast, one for the northeast coast and two for the south coast (see Supplementary Fig. S1 for geodetic monument locations). Measurements were collected at all harbor dock vertices, and at points spaced approximately 2 m apart along dock edges. These data were used to generate interpolated surfaces characterizing individual docks. The resulting surfaces were then shifted horizontally 0.75 m in the direction of 276° from north to correct for an observed, and very consistent, horizontal offset between the coordinates of the established geodetic monuments derived from our survey and those from the 2016 LiDAR survey. This offset can be explained by plate tectonic motions^36^.

A vertical displacement of approximately − 0.01 m due to plate tectonic motion between 2016 and 2025 is expected based on the most recent plate velocity estimates for the SIRGAS station on Rapa Nui^36^. However, the vertical offsets we observed between the elevations among the various geodetic monuments was not consistent. The inconsistent offsets resulted in the interpolated surfaces displaying small local vertical offsets of ± 0.1 m between the dock surfaces and the LiDAR points, likely reflecting localized referencing differences associated with a smaller number of geodetic monuments used during our survey in comparison to the LiDAR survey. Since the LiDAR was collected with a greater number of geodetic monuments, we applied localized vertical shifts to the dock surfaces to align them with the LiDAR data. The magnitude of each adjustment was determined by evaluating the vertical differences across flat areas of the individual docks. The corrected dock surfaces were then overlaid on the LiDAR DEM. A single-value grid, set below the lowest DEM value, was added seaward of the DEM to extend the elevation domain beyond the coastline.

We conducted a vertical error assessment using ground control points distributed along the coast and across different land cover types (the ground control points are a subset of the surveyed elevations taken at coastal *ahu*; see next section). The assessment indicated that the DEM had a vertical bias of approximately 0.19 m. Following the ASPRS Positional Accuracy Standards for Digital Geospatial Data^67^, we performed a vertical adjustment to remove the bias, resulting in an root-mean-square error (RMSE) and standard deviation of error of 0.15 m, closely matching the 0.138 m standard deviation of error reported for the LiDAR survey^66^. Based on this, we define the terrain vertical uncertainty as 0.14 m for modeling purposes. We consider this vertical uncertainty representative of the entire DEM, including the harbor docks as the interpolated surfaces were aligned with the LiDAR points. Finally, the DEM was vertically shifted by -3.215 m (the difference between LMSL and the WGS84 ellipsoid at the tide gauge location) to represent elevations relative to LMSL.

### Coastal *ahu* survey

In January 2025, we surveyed the perimeter of coastal *ahu* using Emlid RS2+ Global Navigation Satellite System receivers. We collected points at the vertices of each *ahu* to derive polygons representing their footprint. If the *ahu* contained collapsed sections, we collected additional points to establish footprints that encompass all of the structure’s constituent parts. We surveyed all *ahu* that are intersected or contained within the 8 m elevation contour line above LMSL. This elevation was determined to ensure the incorporation of all *ahu* that could potentially be reached by the extent of inundation for all proposed SLR scenarios. This 8 m contour line was derived from an existing DEM, created as part of the 2016 LiDAR survey previously mentioned. As with the survey of harbor docks, coastal *ahu* survey points were shifted horizontally 0.75 m in the direction of 276° from north to account for plate tectonic motion between the LiDAR acquisition date and *ahu* survey date. The coastal *ahu* were remotely identified in aerial imagery, collected during the 2016 LiDAR survey, using archaeological maps and site descriptions in the *Atlas Arqueológico de Isla de Pascua*^68^, and books *1000 años en Rapa Nui*^43^, *La Tierra de Hotu Matu’a*^44^ and *Ahu - The Ceremonial Stone Structures of Easter Island*^45^.

### Hydrostatic modeling and inundation analysis

Our modeling of future SLR scenarios adopts a probability-based methodology similar to that employed by NOAA for generating “confidence areas” in inundation mapping^69,70^, which builds upon principles previously described by Gesch (2009)^71^. This methodology assumes that elevation errors associated with the DEM follow a normal distribution and are not systematically biased, allowing the RMSE of the DEM to be considered analogous to the standard deviation of its errors. In this context, NOAA uses a standard score to associate a projected sea level *η* with each DEM pixel elevation *µ* and its uncertainty *σ*, using the equation:

1 $$\:Standard\:Score=\:\frac{\eta\:-\mu\:}{\sigma\:}\:\:\:\:\:\:\:\:\:\:\:\:\:\:\:\:$$

The standard score is used to find its associated percentile rank to determine the likelihood of a location being inundated based on the distance of projected sea level to the DEM pixel elevation and the uncertainty of the elevation data. NOAA uses a one-tailed cumulative percentage to identify the maximum elevation that bounds inundation (all lower elevations are presumed inundated) for a particular confidence level. By establishing a confidence level threshold of 80%, NOAA identifies areas of high probability of inundation.

We follow the NOAA approach and include uncertainty in both the water surface elevation and DEM pixels. We use inundation depth *D* = *W* – *DEM*, where water level (*W*) and DEM elevation (*DEM*) are assumed to be independent, normally distributed random variables. It follows that flood depth (*D*) is also a normally distributed random variable with mean $\:{\mu\:}_{d}={\mu\:}_{w}-{\mu\:}_{DEM}$, and standard deviation (cumulative uncertainty) $\:{\sigma\:}_{d}=\sqrt{{\sigma\:}_{w}^{2}+{\sigma\:}_{DEM}^{2}}$. Thus, the standard score for a prescribed inundation depth (*d*) is expressed as:

2 $$\:Standard\:Score=\:\frac{d-({\mu\:}_{w}-{\mu\:}_{DEM})}{\sqrt{{\sigma\:}_{w}^{2}+{\sigma\:}_{DEM}^{2}}}$$

The probability that inundation depth will exceed a specific depth *d* can then be expressed as:

3 $$\:Pr\left(D>d\right)=1-\varPhi\:\left(\frac{d-({\mu\:}_{w}-{\mu\:}_{DEM})}{\sqrt{{\sigma\:}_{w}^{2}+{\sigma\:}_{DEM}^{2}}}\right)=\:\frac{1}{2}erfc\left(\frac{d-({\mu\:}_{w}-{\mu\:}_{DEM})}{\sqrt{{\sigma\:}_{w}^{2}+{\sigma\:}_{DEM}^{2}}*\sqrt{2}}\right)$$

Where *Φ* is the cumulative distribution of the standard normal and *erfc* is the complementary error function. While we recognize that *Φ* or *erfc* can be used in calculations, we prefer *erfc*. We set *Pr(D > d)* to the desired confidence level, which in our case is 0.8 (80%) and solve for *d* to directly yield the inundation depth that corresponds to a specific confidence level at each DEM pixel location:

4 $$\:d=\:{(\mu\:}_{w}-{\mu\:}_{DEM})+\sqrt{{\sigma\:}_{w}^{2}+{\sigma\:}_{DEM}^{2}}*\:\sqrt{2}*{erfc}^{-1}[2\mathrm{*}0.8]$$

For practical mapping purposes, we classify pixels with a positive value of *d* as flooded, while those with a negative *d* are considered dry. Comparisons of outcomes for different probability values used in confidence mapping are shown in Supplementary Figs. S4–6.

Building on the findings by Habel et al. (2019)^72^, which demonstrate that the groundwater level at high tide in coastal areas can be approximated by the hydrostatic MHHW surface, we depict the extent of flooding distinguishing between areas topographically connected and disconnected to the ocean. Raster grid cells that form a cluster with successively adjacent positive values using the eight-sided connectivity rule tracing to the ocean are considered marine flooding, while those with no continuous path of positive values to the ocean are classified as groundwater inundation^69^. Finally, small groundwater inundation clusters are discarded and classified as dry ground^69^.

To project SLR inundation, we model sea levels in 30 cm increments, starting from present-day MHHW up to 3.9 m of SLR. For each SLR scenario, we also model the 1% AEP level by adding 0.832 m above the SLR increment. Since our ocean surface reference is MHHW, the first scenario represents present-day MHHW and MHHW + 0.832 m; the second includes MHHW + 0.3 m and MHHW + 1.132 m; the third includes MHHW + 0.6 m and MHHW + 1.432 m, and so on, up to MHHW + 3.9 m and MHHW + 4.732 m. Inundation impacts were assessed by overlaying the modeled flooding extents with spatial layers representing the footprint of *ahu* and harbor docks. *Ahu* were considered inundated if the inundation extent intersected any portion of their footprint. Because harbor docks are mostly flat, inundation was classified partial or total based on the proportion of each dock’s planimetric footprint intersected by the inundation extent. Intersections exceeding 10% of the footprint area were classified as partial, whereas those exceeding 90% were classified as total.

### Uncertainties and assumptions

Our analysis accounts for uncertainties in the DEM and the ocean surface elevation, while uncertainties in future sea levels are not considered, as we assume prescribed sea-level increments. Hydrostatic modeling is performed under the assumption that (1) the sea surface level has a single value in the entire domain, (2) tide and other recurring oceanographic dynamics at Rapa Nui do not change in future sea-level scenarios (i.e., MHHW and 1% AEP remain unchanged regardless of the SLR scenario) and (3) coastal morphological changes are not considered. Other assumptions related to probability and statistics are detailed in the *Hydrostatic modeling and inundation analysis* section.

We acknowledge the methodology recommended by Gesch (2018, 2013)^73,74^ for establishing a statistically significant minimum SLR increment based on the vertical uncertainty of the elevation data. In our case, a strict application of Gesch’s methodology would yield a minimum SLR increment between 0.55 m (based only on the DEM’s vertical uncertainty) and 0.72 m (based on the cumulative vertical uncertainty) at 95% confidence. In this context, a SLR increment of 30 cm would fall between the minimum SLR increment calculated with only the DEM’s vertical uncertainty and the cumulative vertical uncertainty at 68% confidence. However, we also recognize the utility of finer SLR increments and recommend that decisions requiring statistical validity with 95% confidence should consider the 0.6 m as the first SLR increments and only every other subsequent increment.

The baseline water level adopted in this study aligns with the most recent IPCC SLR projections^1^, corresponding to the tidal epoch 1995–2014, which differs from the Sweet et al. (2022) baseline^31^, based on the 1991–2010 tidal epoch, by 0.017 m. This discrepancy is negligible in the context of our analysis as it falls well within the cumulative vertical uncertainty of the elevation data.

## Supplementary Information

Below is the link to the electronic supplementary material.

Supplementary Material 1.

## Data Availability

The data used in this study is available from the online repository (https://doi.org/10.5281/zenodo.17517618), the groups in the text or in citations. Other intermediate products are available upon request to npaoakan@hawaii.edu.
