# Supplementary material for: Hydrostatic sea-level rise inundation impacts on ahu and harbors of Rapa Nui (Easter Island)
Source: Sci Rep. 2026 Mar 22;16:14509. doi: 10.1038/s41598-026-45195-9 (PMC13149520; doi:10.1038/s41598-026-45195-9)
Supplement: Supplementary file 1 — Supplementary Material 1. [file 41598_2026_45195_MOESM1_ESM.docx]

Supplementary material for “Hydrostatic sea-level rise inundation
impacts on *ahu* and harbors of Rapa Nui (Easter Island)”

**Noah Paoa^1, *^, Charles H. Fletcher^1^, Matthew Barbee^1^, Tiffany R. Anderson^1^, Shellie Habel^1^, Gabriel Wilkins Riroroko^2^****, Sebastian Pakarati Trengove^3^**

^1^Department of Earth Sciences, School of Ocean and Earth Science and Technology, University of Hawaiʻi at Mānoa, 1680 East‑West Rd., Honolulu, HI, United States. ^2^Departamento de Urbanismo, Municipalidad de Rapa Nui, Atamu Tekena s/n, Rapa Nui, Chile. ^3^Secretaría Técnica de Patrimonio, Hotu Matu’a s/n, Rapa Nui, Chile. *email: [npaoakan@hawaii.edu](mailto:npaoakan@hawaii.edu)

The methodology for this study can be subdivided into three components: (1) characterization of the harbor docks and the footprint of the *ahu*, (2) data processing, and (3) Sea level rise (SLR) modeling and analysis. Section 4 covers uncertainties and assumptions.

*Harbor docks and ahu footprints*

**Existing geospatial datasets**. The study presented in “Hydrostatic sea-level rise inundation impacts on ahu and harbors of Rapa Nui” relies heavily on data collected in a geospatial survey by UASVISION in April and May of 2016. This survey was financed by the Secretaría Técnica de Patrimonio Rapa Nui (STP; Rapa Nui Heritage Technical Secretariat) to support archaeological-related research. Among other outputs, it produced the first LiDAR point dataset and digital elevation model (DEM) of Rapa Nui (1 m resolution), as well as a high-resolution orthomosaic (0.1 m resolution). Data were acquired using a Cessna 172E Monomotor equipped with a Leica ALS60 sensor for LiDAR and an RCD105 camera to capture aerial imagery[1]. The survey report indicates an average flight altitude of 1500 m and an average LiDAR point spacing of 0.25 m. Elevations are orthometric using the EGM 2008 geoid. Unfortunately, the DEM suffers from several issues associated to LiDAR misclassification, particularly along coastal areas, rendering unsuitable for our analysis. The processing steps undertaken to correct the LiDAR and to produce a DEM to meet our requirements are described below.

**Surveying**. Since 2016, several harbors of Rapa Nui had been modified. To accurately characterize the harbor docks and incorporate these changes into the DEM, we conducted a survey in January 2025 using two Emlid RS2+ global navigation satellite system (GNSS) receivers. The receivers operated in a base-rover configuration, communicating over internet for the west coast and via LoRa for the rest of the island. All points were collected using four base station locations. One base station was established in Hanga Roa near an internet source using the Precise Point Positioning technique for approximately 10 hrs (BP01). This data was processed using the Canadian Reference System Precise Point Positioning service (https://natural-resources.canada.ca/maps-tools-publications/data). The remaining three base locations correspond to established geodetic monuments locally known as IP05, IP12, and TM16. The coordinates of these geodetic monuments were determined using the static survey method, following the time-to-distance ratios specified for geodetic surveys in the chapter Especificaciones Técnicas Topográficas y Geodésicas of the Manual de Obras de Vialidad, Pavimentación y Aguas Lluvias (2020)[2] (Topographic and Geodetic Technical Specifications of the Manual of Road Works, Paving and Stormwater) of the Chilean Ministry of Housing and Urban Planning, and processed using Emlid Studio 1.9.

**Harbor docks**. At the harbor docks, we collected all the points necessary to reconstruct their geometry in a virtual environment. The dataset includes points marking all their vertices (including stair steps and terrace-like features), points along all edges at approximately 2 m intervals, and additional points across opposite edges when these were more than 2 m apart. Most points were gathered using the real-time kinematics technique with a few exceptions where internet connectivity and LoRa signal was lost. In such cases, the post-processing kinematics technique was used. Four geodetic monuments were used to survey the harbors; BP01 for Hanga Roa o Tai and Hanga Piko (west coast), IP05 for Hanga Rau and Hanga Ho’onu (northeast coast), IP12 for Hanga Hotu Iti (southeast coast) and TM16 for Hanga Te’e (southeast coast) (Supplementary Fig. S1). Using the surveyed points, we interpolated surfaces representing the harbor docks and integrated the results into the DEM product.

***Ahu***. The objective of the *ahu* survey was to accurately determine their location relative to the coastline as represented on the DEM. Prior to fieldwork, we conducted a visual inspection of the orthomosaic to identify the *ahu* that required surveying. *Ahu* were selected based on their approximate elevation relative to local mean sea level (LMSL). To aid in this selection, we derived an 8 m contour line from the 2016 LiDAR DEM, overlaid it on the orthomosaic, and visually inspected coastline to identify *ahu* footprints intersecting or located within this contour. Supplementary Fig. S1 shows the location and names of all identified *ahu*. The approximate location of the prospective *ahu* was informed by co-author Sebastián Pakarati Trengove, as well as by archaeological maps and site descriptions from the *Atlas Arqueológico de Isla de Pascua*[3], and the books *1000 años en Rapa Nui*[4], *La Tierra de Hotu Matu’a*[5] and *Ahu - The Ceremonial Stone Structures of Easter Island*[6]. In the field, we collected GNSS points at the lower corners of each ahu, where adjoining walls meet, to map their ground-level footprints. For *ahu* with collapsed sections, additional points were collected to ensure the resulting polygons encompass all structural components. Note that Ahu Ora Tu Tu’u (*ahu* no. 67), on the north coast, was unintentionally omitted during the survey planning and identified only later. Although its footprint, derived from the orthomosaic, indicates that it lies at least partially within the 8 m contour line relative to LMSL and therefore should have been surveyed, it was not reached by inundation under any of the modeled SLR scenarios.


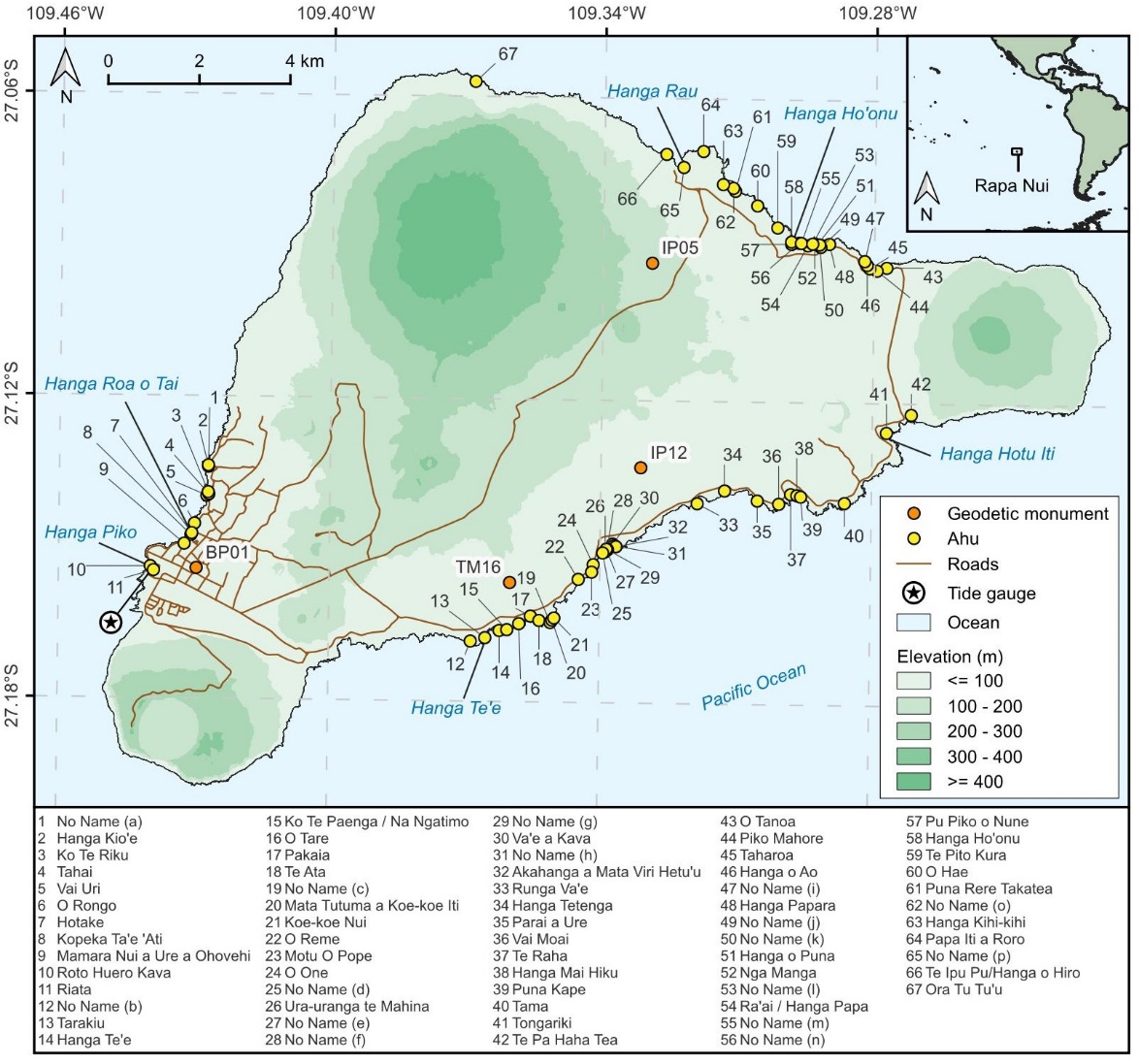


**Supplementary Figure S1**. Geographical context of Rapa Nui, showing the distribution of all surveyed *ahu* and harbors. The inset map identifies Rapa Nui in reference to Central and South America. The locations of *ahu* are identified with yellow circles, geodetic monuments with orange circles, and the tide gauge with a black star. The numbers associated with each yellow circle identify the name of the *ahu*. (Figure created using QGIS 3.40.5; <https://qgis.org/>)

*Data Processing*

**Plate tectonics**. We compared the monument coordinates and ellipsoidal heights measured in January of 2025 with those calculated by UASVISION between April and July of 2016. From these data, we computed the average annual rates of movement in the horizontal and vertical directions relative to the 2016 coordinates. The mean annual rates for the E, N, and vertical components were 0.0865 m yr⁻¹, -0.009 m yr⁻¹, and -0.01 m yr⁻¹, respectively. UASVISION previously reported rates of 0.0686 m yr⁻¹ E, -0.0065 m yr⁻¹ N and -0.0023 m yr⁻¹ H [1]. These values are consistent with those from the SIRGAS-CON geodetic station ISPA on Rapa Nui, which reports 0.0664 +/- 0.00033 m yr⁻¹ E, -0.0058 +/- 0.0004 m yr⁻¹ N, and -0.00137 +/- 0.0005 m yr⁻¹ H [7]. Our mean horizontal rates of movement are comparable to those from both UASVISION and ISPA station. Moreover, the coordinate difference between 2016 and 2025 at the geodetic monuments were highly consistent at approximately 0.75 m toward 96° from north. Accordingly, we shifted all the surveyed points 0.75 m toward 276° from north to align them with the LiDAR data.

Unlike the horizontal movement, the vertical component showed greater inconsistency. The calculated mean annual rate of vertical movement was nearly an order of magnitude lower than the UASVISION and ISPA station values. These discrepancies produced localized offsets between the interpolated harbor dock surfaces and the LiDAR points cloud, typically within ±0.1 m, making it difficult to seamlessly merge the two data. Therefore, we applied localized vertical displacements of ±0.1 m to the surfaces in order to better align them with the LiDAR points.

**LiDAR processing**. To facilitate the LiDAR processing, we used the original 2016 LiDAR DEM to generate a contour line at 10 m above LMSL, with an additional 2 m horizontal landward buffer. This contour was used to select the LiDAR points to be processed. Along several coastal areas outside the harbor, we observed noticeable misclassified points. Some appeared to represent birds or low clouds, but the majority were likely caused by variations in tidal levels during different data collection times, as well as wave splashes and whitewash. The latter two were expected, given the large waves and extensive whitewash visible in the orthomosaic. To correct these issues, we manually reclassified points using 2-5 m cross-shore profiles. Because our objective was to create a bare-earth DEM, the classification was simplified into two categories: ground and non-ground points. Offshore water points captured at high tide were easily identified, appearing as horizontal point alignments above sloping ones (Supplementary Fig. 2c) or several stacked. Similarly, splashes were recognizable as high, spare outliers (Supplementary Fig. 2a). Additional misclassifications were observed in tide pools filled during wave run-up, appearing as flat horizontal point alignments over concave or jagged terrain. In these cases, most points were removed, retaining only the lower ones.


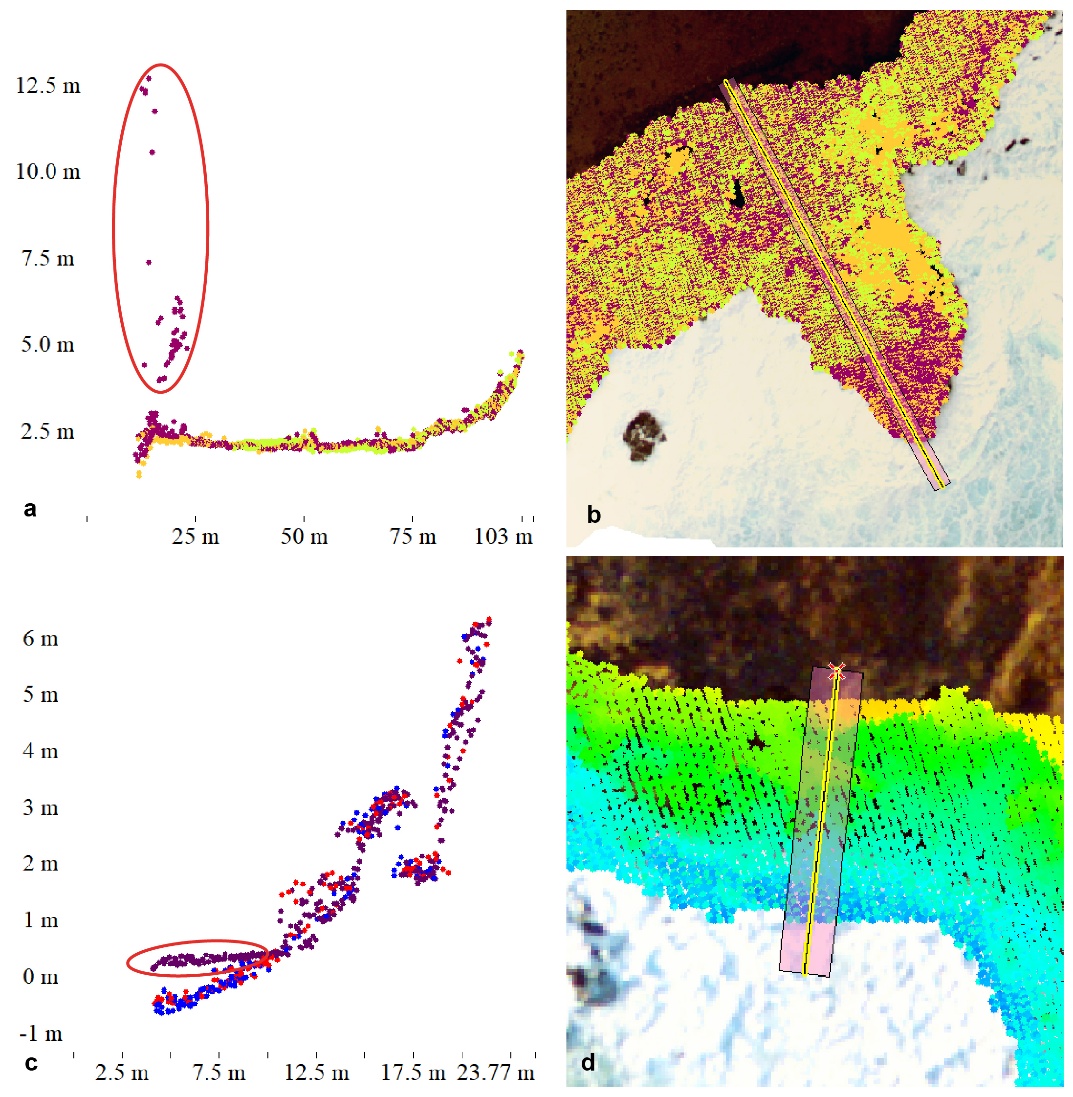


**Supplementary Figure S2**. Examples of commonly misclassified LiDAR points. **a** and **b** illustrate wave splash, and **c** and **d** illustrate differing tides or wave run up captured during different collection times.

Once the coastline was cleaned, we removed vegetation, buildings, and other anthropogenic structures to produce a bare-earth DEM. *Ahu* were preserved as they often seamlessly blend into the surrounding landscape, making it difficult to distinguish between the man-made structure and the underlying topography. Similarly, many rock formations were retained, as differentiating between surface rocks and lava flow outcrops on the aerial imagery was difficult. From the cleaned point cloud, we generated a 1 m raster grid using the “triangulated irregular network” method in Global Mapper 24.1. EGM 2008 geoid values were subtracted to obtain WGS84 ellipsoidal elevations, matching the vertical reference of the harbor dock surveys. The harbor surfaces were overlayed on the cleaned DEM and a single-value surface grid, set below the lowest DEM value, was applied seaward of the DEM to extend the elevation domain beyond the coastline and facilitate modeling.

**Vertical assessment.** To assess the vertical accuracy of the final DEM, we selected all surveyed points that, based on the orthomosaic, appeared to have been collected on relatively flat terrain. This criterion excluded points located near steep *ahu* walls or cliffs, as such areas are prone to producing large elevation differences that are more likely associated to the resolution of the DEM rather than the elevation accuracy of the input data. Following the ASPRS Positional Accuracy Standards for Digital Geospatial Data[8], we sampled DEM elevations at the locations of the selected points and calculated the mean error, and standard deviation and root mean square error (RMSE) of the differences. The initial results showed a mean error of -0.14 m and a RMSE nearly twice the standard deviation, suggesting the presence of bias in the data[8]. The biases were treated by lowering the DEM by the amount of the mean error[8]. Repeating the assessment on the adjusted DEM yielded a standard deviation and RMSE of 0.14 m, which was used as the vertical uncertainty of the DEM for modeling purposes.

*SLR modeling and analysis*

The SLR modeling procedures and analysis of results are described in detail in the main manuscript. SLR inundation layers at mean higher high water (MHHW) and the 1% annual exceedance probability (AEP) events were generated for the entire island across all SLR scenarios, ranging from 0 to 3.9 m in 0.3 m increments. Supplementary Figure S3 shows examples of these layers at the harbor locations. All layers and results can be made available to support community and local government efforts in planning and developing adaptation strategies. Differences between the confidence used in the probability-based mapping is illustrated in Supplementary Figs. S4-6.

**
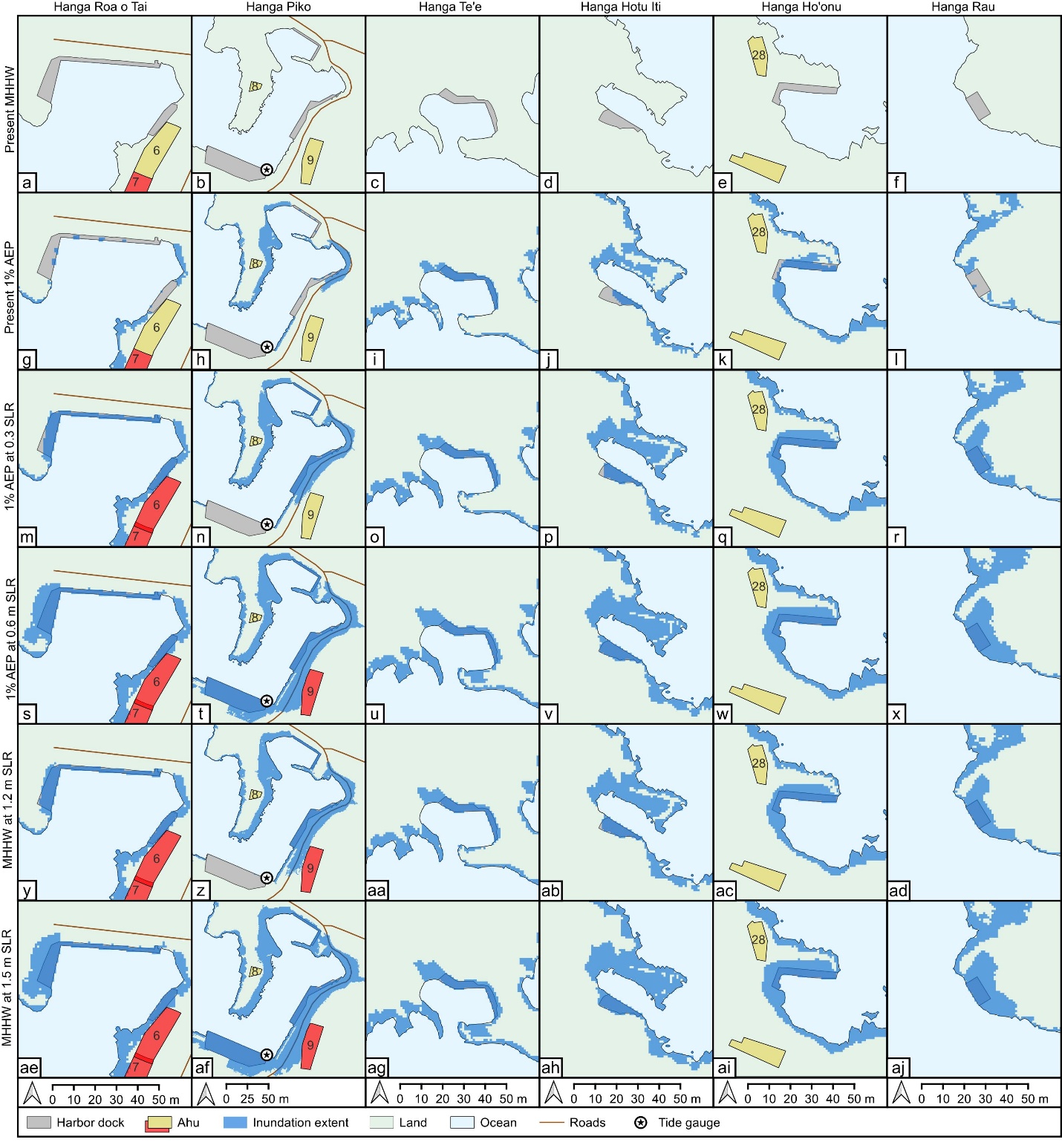
Supplementary Figure S3**. SLR inundation extent at Rapa Nui harbors. Rows correspond to specific SLR scenarios and columns correspond to individual harbors. *Ahu* are shown as yellow and red polygons depending on whether the *ahu* are intersected by the inundation or not, respectively. Harbors docks are represented as grey polygons. (Figure created using QGIS 3.40.5; <https://qgis.org/>)


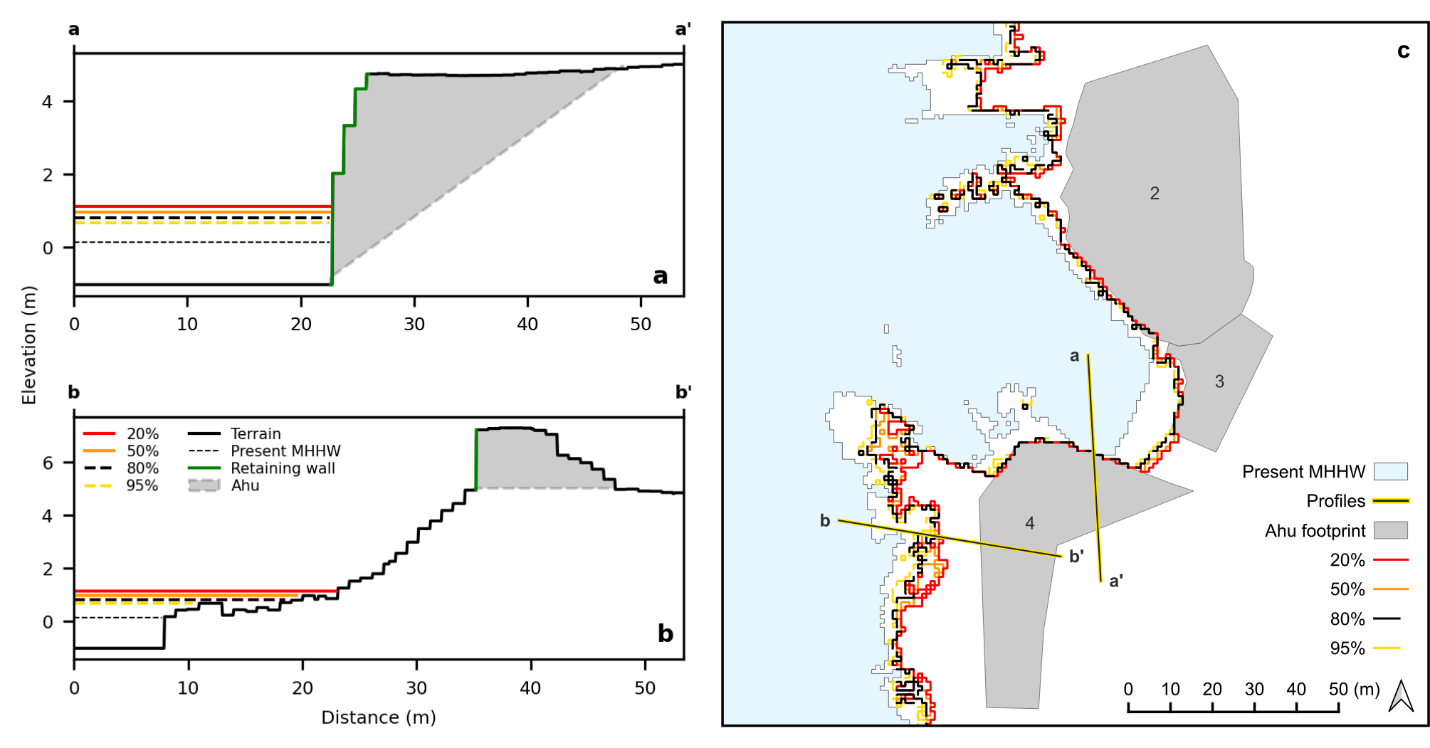


**Supplementary Figure S4**. Comparison of confidence levels used in probability-based mapping for the 1% AEP level at 0 m of SLR. Panels **a** and **b** show cross-sections, and panel **c** presents a map view. In all panels, probability values of 20%, 50%, 80% and 95% are shown as a red, orange, black dashed and yellow dashed lines, respectively. Present-day MHHW (always at 80%) is represented by a thin black dashed line in panels **a** and **b**, and by the blue area panel **c**. In the cross-sections, the black line denotes terrain elevation, the green line indicates the *ahu* retaining walls and the grey shaded area represents the *ahu* structure, with a dashed base denoting unknown underlying terrain. (Panel **c** created using QGIS 3.40.5; <https://qgis.org/>)


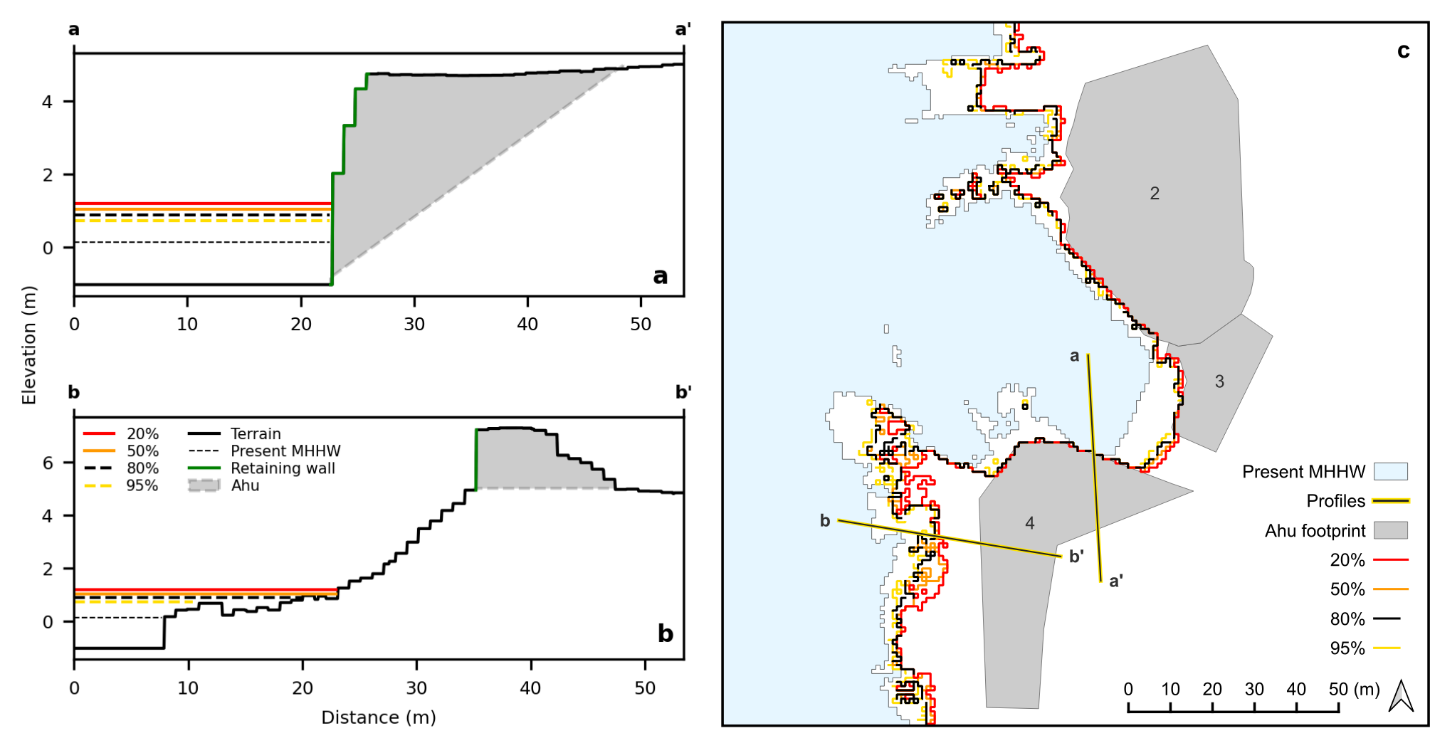


**Supplementary Figure S5**. Comparison of confidence levels used in probability-based mapping for the MHHW level at 0.9 m of SLR. Panels **a** and **b** show cross-sections, and panel **c** presents a map view. In all panels, probability values of 20%, 50%, 80% and 95% are shown as a red, orange, black dashed and yellow dashed lines, respectively. Present-day MHHW (always at 80%) is represented by a thin black dashed line in panels **a** and **b**, and by the blue area panel **c**. In the cross-sections, the black line denotes terrain elevation, the green line indicates the *ahu* retaining walls and the grey shaded area represents the *ahu* structure, with a dashed base denoting unknown underlying terrain. (Panel **c** created using QGIS 3.40.5; <https://qgis.org/>)


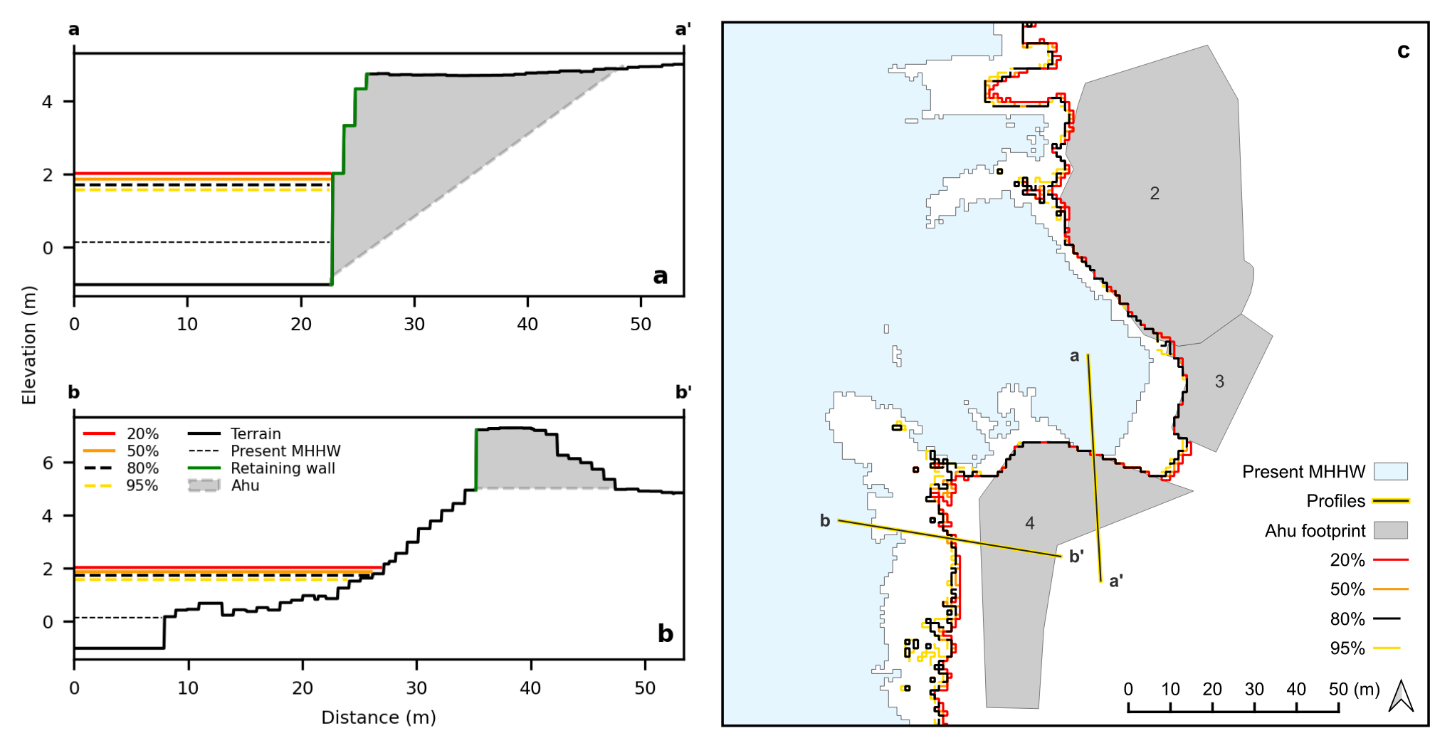


**Supplementary Figure S6**. Comparison of confidence levels used in probability-based mapping for the 1% AEP level at 0.9 m of SLR. Panels **a** and **b** show cross-sections, and panel **c** presents a map view. In all panels, probability values of 20%, 50%, 80% and 95% are shown as a red, orange, black dashed and yellow dashed lines, respectively. Present-day MHHW (always at 80%) is represented by a thin black dashed line in panels **a** and **b**, and by the blue area panel **c**. In the cross-sections, the black line denotes terrain elevation, the green line indicates the *ahu* retaining walls and the grey shaded area represents the *ahu* structure, with a dashed base denoting unknown underlying terrain. (Panel **c** created using QGIS 3.40.5; <https://qgis.org/>)

*References*

1. Castillo Iturrieta, C. & Rojas Reyes, F. *INFORME TÉCNICO LEVANTAMIENTO LIDAR*. www.uasvision.cl (2016).

2. Ministerio de Vivienda y Urbanismo. Cap. 6 Especificaciones Técnicas Topográficas y Geodésicas. in *Manual de Obras de Vialidad, Pavimentación y Aguas Lluvias* (2020).

3. Cristino Ferrando, C., Vargas Casanova, P. & Izaurieta San Juan, R. *Atlas Arqueológico de Isla de Pascua*. (Corporación Toesca: Facultad de Arquitectura y Urbanismo, Universidad de Chile, Santiago, 1981).

4. Vargas, P., Cristino, C. & Izaurieta, R. *1000 Años En Rapa Nui*. (Editorial Universitaria, S.A., 2006).

5. Englert, S. *La Tierra de Hotu Matu’a*. (San Francisco, 1948).

6. Martinsson-Wallin, H. *Ahu—The Ceremonial Stone Structures of Easter Island*. (Societas Archaeologica Upsaliensis, 1994).

7. Sánchez, L., Drewes, H., Kehm, A. & Seitz, M. SIRGAS reference frame analysis at DGFI-TUM. *Journal of Geodetic Science* **12**, 92–119 (2022).

8. Abdullah, Q., Munjy, R., Nimetz, J., Zoltek, M. & Lee, C. *Positional Accuracy Standards for Digital Geospatial Data*. (ASPRS, 2024).
